# Effects of sonication parameters on transcranial focused ultrasound brain stimulation in an ovine model

**DOI:** 10.1371/journal.pone.0224311

**Published:** 2019-10-24

**Authors:** Kyungho Yoon, Wonhye Lee, Ji Eun Lee, Linda Xu, Phillip Croce, Lori Foley, Seung-Schik Yoo

**Affiliations:** 1 Department of Radiology, Brigham and Women’s Hospital, Harvard Medical School, Boston, Massachusetts, United States of America; 2 Translational Discovery Laboratory, Brigham and Women’s Hospital, Harvard Medical School, Boston, Massachusetts, United States of America; University of Toronto, CANADA

## Abstract

Low-intensity focused ultrasound (FUS) has significant potential as a non-invasive brain stimulation modality and novel technique for functional brain mapping, particularly with its advantage of greater spatial selectivity and depth penetration compared to existing non-invasive brain stimulation techniques. As previous studies, primarily carried out in small animals, have demonstrated that sonication parameters affect the stimulation efficiency, further investigation in large animals is necessary to translate this technique into clinical practice. In the present study, we examined the effects of sonication parameters on the transient modification of excitability of cortical and thalamic areas in an ovine model. Guided by anatomical and functional neuroimaging data specific to each animal, 250 kHz FUS was transcranially applied to the primary sensorimotor area associated with the right hind limb and its thalamic projection in sheep (n = 10) across multiple sessions using various combinations of sonication parameters. The degree of effect from FUS was assessed through electrophysiological responses, through analysis of electromyogram and electroencephalographic somatosensory evoked potentials for evaluation of excitatory and suppressive effects, respectively. We found that the modulatory effects were transient and reversible, with specific sonication parameters outperforming others in modulating regional brain activity. Magnetic resonance imaging and histological analysis conducted at different time points after the final sonication session, as well as behavioral observations, showed that repeated exposure to FUS did not damage the underlying brain tissue. Our results suggest that FUS-mediated, non-invasive, region-specific bimodal neuromodulation can be safely achieved in an ovine model, indicating its potential for translation into human studies.

## Introduction

Non-invasive functional modulation of region-specific cortical/subcortical activity is anticipated to offer new non-pharmacological treatments of neurological and psychiatric disorders [1, 2], as well as new tools for functional brain mapping [3–5]. Brain stimulation techniques, such as transcranial direct current stimulation (tDCS) and transcranial magnetic stimulation (TMS), have shown efficacy for ameliorating neuropsychiatric diseases [6–8]. However, these approaches, which are based on application of electrical currents or electromagnetic waves, generally lack spatial specificity of modulatory area on the order of centimeters and penetration depth using these methods is limited to the cortical surface [9, 10]. Optogenetic approaches provide cellular-scale modulation of neuronal activity [11] but require genetic modification of target neural cells to gain light-sensitive excitability, thus limiting translation into clinical practice.

Focused ultrasound (FUS) technology allows for non-invasive delivery of acoustic pressure waves to a localized area of biological tissue with greater spatial selectivity, measuring only a few millimeters in diameter, and with the ability to reach deep tissue regions for thermal and non-thermal therapies, such as hyperthermic ablation or lithotripsy [12–15]. Advancement in FUS techniques has enabled highly-focused transcranial delivery of ultrasound to specific brain regions using independent actuation of multiple arrays of ultrasound transducers [16–18] or a single-element FUS transducer [4, 19]. Since the pioneering works demonstrating that application of acoustic pressure waves can reversibly change neural excitability [20], an increasing number of studies, especially during the past decade, have shown that non-thermal, low-intensity FUS can alter regional neural activity in both the central and peripheral nervous systems [4, 21–28]. The previous investigations have revealed that the neuromodulatory effects on neuronal tissue are bimodal, i.e., suppressive or excitatory depending on the choice of pulsing schemes [4, 28–30]. Altogether, these efforts have facilitated the emerging field of FUS-mediated non-invasive neuromodulation.

The sonication parameters, and particularly the pulsing schemes, are known to determine the stimulation efficiency, as shown in prior research primarily utilizing small animal models such as rodents or rabbits [4, 28–30]. Studies have also been performed in sheep [31] and pigs [32] as well as in non-human primates [33–36], and exploratory studies have been reported using healthy human volunteers [37–44] and an individual with impaired consciousness [45]. Nonetheless, further investigation examining the effect of varying sonication parameters on neuromodulation in a large animal model is needed to provide important translational information for this technique, including its safety.

We previously reported our investigation on the effects of FUS on stimulation of the sensorimotor and visual cortices in an ovine model [31]. We chose to study sheep due to the structural similarities between the sheep and human craniums in regards to thickness, radius of curvature, and porosity [46, 47] and the neuroanatomical structures that are non-homogeneous and gyrencephalic [48]. Moreover, available clinical models of epilepsy [49], stroke [50], and brain injury [51] make sheep an attractive species to study *en route* to human application. However, systematic assessment of sonication parameters, especially regarding pulsing schemes, has not been performed and warrants further probing.

In the present study, we examined the effect of varying FUS sonication parameters on the excitation and suppression of region-specific cortical and deep (thalamic) brain regions in sheep. The animal’s primary motor (M1) and sensory cortices (S1) of the unilateral (right) hind leg, as well as the corresponding thalamic structures of ventrolateral nucleus (VL) mediating the motor efferent pathway and ventral posterolateral nucleus (VPL) mediating the sensory afferent pathway, were identified using functional magnetic resonance imaging (fMRI). As guided by anatomical and functional MRI data, FUS was transcranially applied to stimulate the identified motor circuit and (in separate sessions) to suppress activity of the sensory areas (i.e., S1 and thalamus) using different sonication parameters, focusing on burst duration, duty cycle, and acoustic intensity. The presence of stimulation of the motor circuits was assessed by electromyography (EMG), while the degree of suppression was assessed by measuring the change in electroencephalography (EEG)-based somatosensory evoked potentials (SEPs) elicited by electrical stimulation of the right hind limb. We conducted post-sonication behavior monitoring as well as MRI and histological analysis performed at variable time points after sonication to evaluate safety and biological effects of repeated FUS sessions.

## Materials and methods

### Animal preparation

All animal procedures were conducted under approval from and according to the regulations and standards of the Institutional Animal Care and Use Committee (IACUC) of the Brigham and Women’s Hospital (Protocol Number: 2016N000074). Only female sheep (Polypay, n = 10, weight = 49.1 ± 4.4 kg, labeled as ‘SH1’ to ‘SH10’) were used in this study, as males may grow scurs (incompletely developed horns) that impede acoustic transmission. The animals were initially sedated using intramuscular (IM) xylazine (0.1 mg/kg), followed by Telazol (mixture of tiletamine and zolazepam, dose of 2–4 mg/kg; additional dose as needed) prior to all experimental procedures. The sheep were intubated to prevent bloating and to assist with respiration under anesthesia. Additional doses of intravenous (IV) Telazol were periodically given to maintain an adequate plane of anesthesia throughout the procedures, based on constant monitoring of end-tidal carbon dioxide (CO_2_; V9004, SurgiVet, Norwell, MA), peripheral oxygen saturation (SpO_2_; V3404P, SurgiVet), and heart rate (3150 MRI Patient Monitor, Invivo Research Inc., Orlando, FL). The assessment of responses to hoof pinching and eyelid touching was also performed before the beginning of each FUS administration to validate the depth of anesthesia. The additional anesthetics were given before/after an experimental block as necessary, but not during administration of FUS sonication. We also avoided administering additional anesthesia during the acquisition of EEG/EMG signals.

### MRI for transcranial FUS navigation

The M1 and S1 areas in the left hemisphere corresponding to sensorimotor stimulation of the contralateral (right) hind leg, as well as their thalamic projections (noted as ‘Thal’; i.e., a thalamic area approximating the locations of VL and VPL), were chosen as sonication targets (see ***‘*Sonication targets and parameters*’*** below) [52–55]. Structural and functional neuroimaging were performed using a 3 Tesla MRI scanner (Signa HDxt, GE Medical Systems, Waukesha, WI) to acquire volumetric MRI data for the later image-guided navigation. Foam padding was applied around the head of the sheep to restrict head movement in an eight-channel phased array head coil. T_1_-weighted high-resolution images covering the entire head were obtained using the inversion recovery 3D spoiled gradient recalled (SPGR) sequence (field of view 25 × 25 cm^2^, slice thickness 1 mm, image matrix 256 × 256, number of slices 156, voxel size 0.98 × 0.98 × 1 mm^3^, repetition time (TR)/echo time (TE) = 7.0/3.1 ms, flip angle 11°). This high-resolution volumetric T_1_ data was used later for image-guided FUS navigation.

To identify the targeted sonication areas in each animal, fMRI was performed using a T_2_*-weighted, blood oxygenation level dependent (BOLD) contrast sensitive gradient-echo echo-planar-imaging (EPI) sequence (field of view 18 × 18 cm^2^, slice thickness 3 mm, image matrix 64 × 64, number of slices 20, voxel size 2.81 × 2.81 × 3 mm^3^, TR/TE = 2,000/40 ms, flip angle 90°). During image acquisition, the right hind leg (near the junction of the lateral gastrocnemius muscle and peroneus muscle) was mechanically stimulated (gentle squeeze at ~2 Hz) for a period of 20 s three times interleaved by four resting periods of the same duration. Previous studies of ours and others have shown that passive actuation of the peripheral muscles activates the corresponding M1 and S1 without volitional control [31, 56–58]. Additionally, for the same volume coverage as the fMRI, T_1_-weighted anatomical images were acquired using a spin echo (SE) sequence (field of view 18 × 18 cm^2^, slice thickness 3 mm, image matrix 512 × 512, number of slices 20, voxel size 0.35 × 0.35 × 3 mm^3^, TR/TE = 400/11 ms, flip angle 65°), and T_2_-weighted images were acquired using a fast spin echo (FSE) sequence (field of view 18 × 18 cm^2^, slice thickness 3 mm, image matrix 512 × 512, number of slices 20, voxel size 0.35 × 0.35 × 3 mm^3^, TR/TE = 3,334/102 ms, echo train length 24). These additional T_1_ and T_2_ data were used to register fMRI data to the volumetric T_1_-weighted SPGR image covering the entire head.

**Fig 1:**
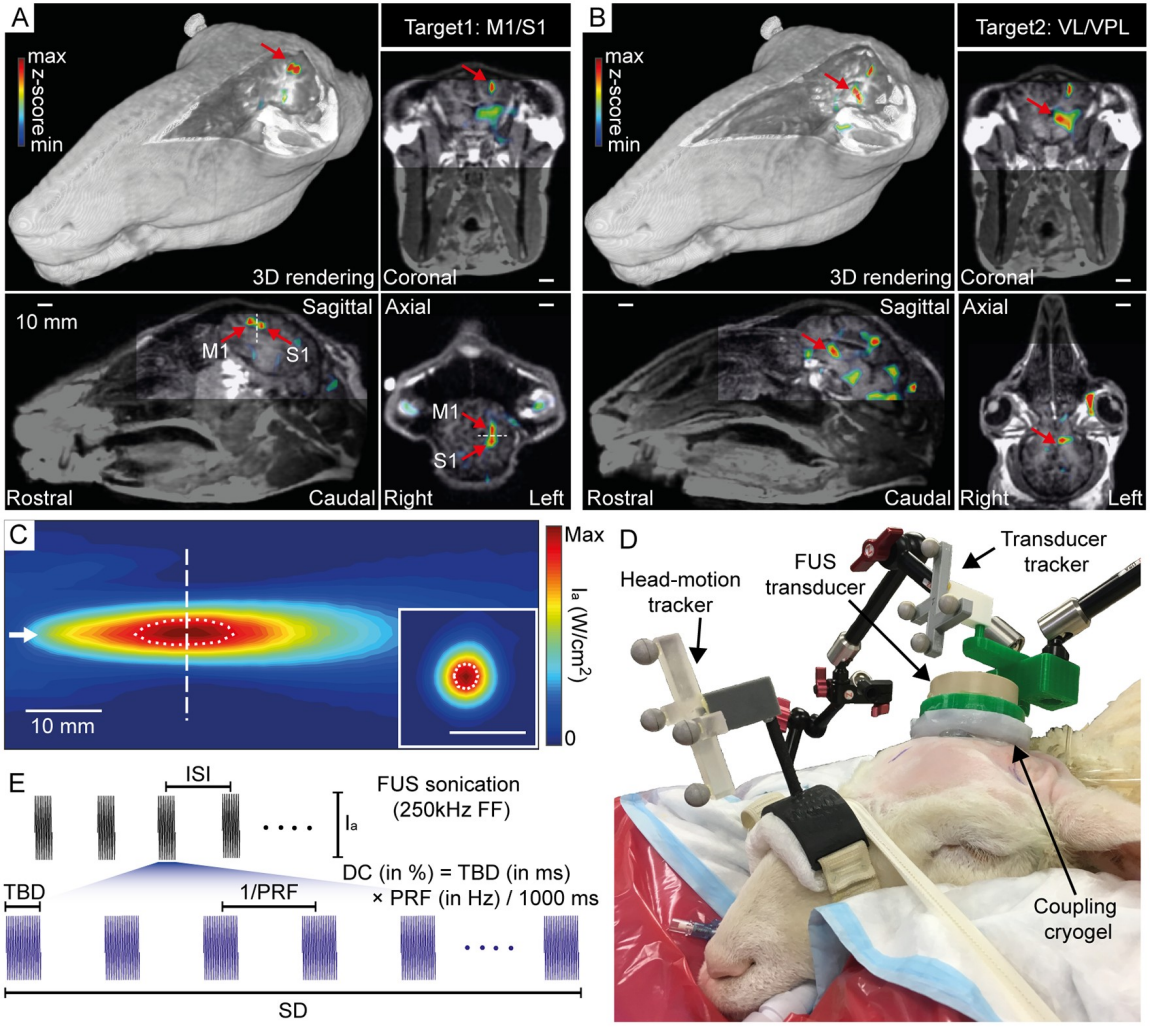
Schematic of the experimental setup. (A) An exemplar 3D rendering and triplanar view of the processed fMRI results (p < 0.05, z-score > 1.64; z-score map in pseudo color) overlaid on the 3D anatomical neuroimaging data that describes locations of the M1 and S1 as well as (B) the VL/VPL, marked by red arrows. The activation loci for the M1/S1 were observed anterior and posterior to the central sulcus (dotted white lines on the sagittal and axial views) whereas the VL/VPL areas did not show distinctive boundaries. (C) Acoustic intensity profiles in the longitudinal and transverse planes. The sonication direction is represented by the short white arrow. The dotted white circle and ellipse indicate the region of 90%-maximum of the intensity profile. (D) Apparatus of the image-guided transcranial FUS setup. (E) Illustration of acoustic parameters: acoustic intensity (I_a_), tone-burst duration (TBD), pulse repetition frequency (PRF), duty cycle (DC), sonication duration (SD), and inter-stimulation interval (ISI). ISI is not applicable for suppressive sonication as a single, 2 min-long sonication was given per session. Scale bar = 10 mm in (A–C, C inset).

The fMRI data was processed through a general linear model (GLM) after applying motion correction and a Gaussian smoothing kernel with full-width at half-maximum (FWHM) of 6 × 6 × 6 mm^3^ using the SPM8 software package (Wellcome Department of Imaging Neuroscience, University College London, London, UK; www.fil.ion.ucl.ac.uk/spm), creating a voxel-wise statistical parametric map with respect to the task-specific canonical hemodynamic response function (HRF). For the visualization of sheep-specific functional activation from the fMRI data, we used *t*-contrast, p < 0.05, z-score > 1.64 without threshold correction. The processed fMRI data was co-registered to the volumetric T_1_-weighted SPGR image using the normalized mutual information technique [59]. Then, the co-registered T_1_ and fMRI images were saved in the digital imaging and communications in medicine (DICOM) format and displayed using the in-house neuro-navigation software [60] (an example of ‘SH7’ is displayed in Fig 1A and 1B).

### Sonication setup and transducer characterization

A single-element FUS transducer (GPS200-400128, Ultran Group, Hoboken, NJ) operating at a fundamental frequency of 250 kHz was used for transcranial delivery of ultrasound. The 37 mm diameter piezo-element disc was coupled with an acoustic lens with a 20 mm radius-of-curvature. The input electrical sinusoidal waveform for the transducer was generated using two serially-connected function generators (33500B, Keysight, Santa Rosa, CA) and was amplified by a linear power amplifier (240L, Electronics and Innovations, Rochester, NY). An impedance matching circuit (JT-800, Electronics and Innovations) was used between the transducer and the amplifier.

Because the focal location and dimension of the transducer is determined by acoustic propagation through the acoustic lens, the spatial profile of the acoustic field from the transducer was mapped in a degassed water tank using a needle-type hydrophone (HNC200, Onda, Sunnyvale, CA) mounted to a three-axis robotic stage (Bi-Slides, Velmex, Bloomfield, NY), covering a longitudinal plane (30 × 70 mm^2^ with 1 mm step) along the beam path and a transverse plane (30 × 30 mm^2^ with 1 mm step) perpendicular to the beam path, as shown in Fig 1C. The area of the neuromodulatory FUS focus, defined as full-width at 90%-maximum area of the acoustic intensity map [61, 62], was 3 mm in diameter and 13 mm in length (indicated by the white dotted circular profiles in Fig 1C). The center of maximum intensity was located 30 mm from the exit plane of the transducer. The acoustic intensity at the FUS focal point with respect to the magnitude of input voltage was separately measured using a calibrated hydrophone (HNR500, Onda).

### Image-guided transcranial FUS setup

For sonication, the animal was positioned prone on the procedure table, and the head (positioned over a foam cushion) was restrained to the table using fabric tape to minimize head movement during sonication (shown in Fig 1D). Using a custom-built neuro-navigation system (details are described in our previous works [31, 60]), the actual space of the sheep’s head and the virtual space of the volumetric T_1_-weighted image were co-registered by matching the coordinates of five anatomical landmarks (inner canthus of the eyes, bottom of the external auditory canal of the ears, nose tip) using an optical pointer tool and infrared tracking camera (Polaris Vicra, Northern Digital Inc., Waterloo, ON, Canada). The quality of co-registration was assessed by the fiducial registration error (FRE) [63]. The FRE was 5.8 ± 1.5 mm for a total of 66 sonication sessions across the ten animals.

Prior to the sonication procedures, the wool over the sheep’s scalp was shaved using electric trimmers and household razors. The FUS transducer was placed over the head of the sheep with a compressible hydrogel (polyvinyl alcohol-PVA, 363065, Millipore Sigma, St. Louis, MO; 7–9% weight per volume in degassed water, two freeze-thaw cycles) [64] and generic ultrasound gel (Aquasonic, Parker Laboratories, Fairfield, NJ) to acoustically couple the FUS transducer and the skin. The PVA hydrogel was used as a compressible stand between the transducer surface and the skin with two different thicknesses, which allowed for selective placement of the acoustic focus on the cortical or thalamic areas using the FUS transducer with a fixed focal geometry. In detail, the PVA hydrogels were prepared with thicknesses of 20 mm (for M1/S1 stimulation) and 5 mm (for thalamic stimulation), measured from the tip of the hydrogel to the exit plane of the transducer. The 20-mm and 5-mm thick coupling gels were compressible to 7-mm and 1-mm, respectively. Therefore, the FUS transducer with 30 mm focal depth (from the exit plane) was used to sonicate a localized brain region either ~10–23 mm or ~25–29 mm deep from the scalp using the coupling gels. The location of the acoustic focal point and corresponding sonication path with respect to the sheep’s head was visualized on the volumetric MRI data *via* spatial information acquired from the transducer tracker (using four infrared-reflective markers, in Fig 1D). Head movement was also independently tracked using a head-motion tracker (mounted over the snout, in Fig 1D). After the FUS focus was targeted to the neuroanatomical region-of-interest based on the planned sonication path, the transducer was locked in place using a swivel ergonomic arm (Zacuto, Chicago, IL) connected to the procedure table.

### Sonication targets and parameters

Bimodal neuromodulatory effects of ultrasound sonication (i.e., excitation and suppression) were separately assessed in different experimental sessions (66 sonication sessions, with a time gap between sessions of 9.8 ± 6.3 days). For assessment of excitatory effects, the excitatory FUS was delivered to the left M1 anterior to the central sulcus (CS) or to the left thalamic region (Thal), while examining the elicited bilateral EMG signals from the hind limbs. For assessment of suppressive effects, the suppressive FUS was delivered to the left S1 posterior to the CS or to the left thalamus, while measuring the SEP EEG induced by simultaneous external electrical stimulation of the right hind limb. The proximity of the VL and VPL in ovine neuroanatomy (example shown in Fig 1B) was beyond the spatial accuracy of sonication (3 mm acoustic focal size in diameter) confounded by the image-registration error (FRE of 5.8 ± 1.5 mm); therefore, a single spatial coordinate in the thalamus was assigned as a sonication target. The distances between M1 and other sonication targets (S1 and thalamus) were 7.6 ± 1.3 mm (n = 10) and 29.6 ± 3.0 mm (n = 10), respectively.

The different sets of excitatory and suppressive sonication parameters used in this study are tabulated in Tables 1 and 2, respectively, with corresponding mechanical index (MI) and number of animals. The components of the parameters are acoustic intensity (I_a_), fundamental frequency (FF), tone-burst duration, duty cycle (DC) with corresponding pulse repetition frequency (PRF), and inter-stimulus interval (ISI) (graphical illustration shown in Fig 1E). These parameter sets were chosen based on our previous investigations in small (rabbits and rats) and large animals (sheep) [4, 29–31, 65], in which a shorter sonication duration (≤ ~500 ms) at a higher DC (≥ 30%) favored excitation, and a longer sonication duration (≥ ~1 min) at a lower DC (≤ 10%) resulted in suppression. We note that two of the animals (‘SH1’ and ‘SH2’) were used in preparing the experimental setup and therefore lacked the systemic application of all sonication parameter sets.

**Table 1:** Combinations of sonication parameters used for examining the excitatory effects. DC = duty cycle, TBD = tone-burst duration, PRF = pulse repetition frequency, SD = sonication duration, I_a_ = acoustic intensity, I_sppa_ = spatial-peak pulse-average intensity, I_spta_ = spatial-peak temporal-average intensity, I_spta_ = I_sppa_ × DC, MI = mechanical index.

Parameter set ID	DC (%)	TBD (ms)	PRF (Hz)	SD (ms)	*In situ* I_a_ (I_sppa_ W/cm^2^)	*In situ* I_a_ (I_spta_ W/cm^2^)	*In situ* MI	Number of animals	
								M1	Thal
	Pulsed mode sonication								
EP1	30	0.5	600	200	15.8	4.7	1.37	8	8
EP2	30	0.5	600	200	18.2	5.5	1.47	8	8
EP3	30	1	300	200	15.8	4.7	1.37	8	8
EP4	30	1	300	200	18.2	5.5	1.47	8	8
EP5	30	2	150	200	15.8	4.7	1.37	8	8
EP6	30	2	150	200	18.2	5.5	1.47	8	8
EP7	30	3	100	200	15.8	4.7	1.37	8	8
EP8	30	3	100	200	18.2	5.5	1.47	8	8
EP9	50	0.5	1000	200	15.8	7.9	1.37	8	8
EP10	50	0.5	1000	200	18.2	9.1	1.47	8	8
EP11	50	1	500	200	15.8	7.9	1.37	9	8
EP12	50	1	500	200	18.2	9.1	1.47	9	8
EP13	50	2	250	200	15.8	7.9	1.37	9	8
EP14	50	2	250	200	18.2	9.1	1.47	9	8
EP15	50	3	167	200	15.8	7.9	1.37	8	8
EP16	50	3	167	200	18.2	9.1	1.47	8	8
EP17	70	0.5	1400	200	15.8	11.1	1.37	8	8
EP18	70	0.5	1400	200	18.2	12.7	1.47	8	8
EP19	70	1	700	200	15.8	11.1	1.37	8	8
EP20	70	1	700	200	18.2	12.7	1.47	8	8
EP21	70	2	350	200	15.8	11.1	1.37	8	8
EP22	70	2	350	200	18.2	12.7	1.47	9	8
EP23	70	3	233	200	15.8	11.1	1.37	8	8
EP24	70	3	233	200	18.2	12.7	1.47	8	8
	Continuous mode sonication								
EP25	100	-	-	60	15.8	15.8	1.37	8	8
EP26	100	-	-	60	18.2	18.2	1.47	8	8
EP27	100	-	-	100	15.8	15.8	1.37	8	8
EP28	100	-	-	100	18.2	18.2	1.47	8	8
EP29	100	-	-	140	15.8	15.8	1.37	8	8
EP30	100	-	-	140	18.2	18.2	1.47	8	8

**Table 2:** Combinations of sonication parameters used for examining the suppressive effects.

Parameter set ID	DC (%)	TBD (ms)	PRF (Hz)	*In situ* I_a_ (I_sppa_ W/cm^2^)	*In situ* I_a_ (I_spta_ W/cm^2^)	*In situ* MI	Number of animals	
							S1	Thal
SP1	5	0.5	100	5.4	0.27	0.80	9	8
SP2	5	0.5	100	11.6	0.58	1.17	10	10
SP3	3	0.5	60	5.4	0.16	0.80	8	8
SP4	3	0.5	60	11.6	0.35	1.17	10	8
SP5	5	1.0	50	5.4	0.27	0.80	8	8
SP6	5	1.0	50	11.6	0.58	1.17	10	8
SP7	3	1.0	30	5.4	0.16	0.80	8	8
SP8	3	1.0	30	11.6	0.35	1.17	8	8

In more detail, for the excitatory sonication in pulsed mode, we used a sonication duration of 200 ms. We combined four tone-burst durations (0.5, 1, 2, and 3 ms), three DCs (30, 50, and 70%, and corresponding PRFs), and two levels of *in situ* I_sppa_ (15.8 and 18.2 W/cm^2^) for a total of 24 parameter sets (named EP1–EP24, see Table 1). Six additional sets of excitatory sonication parameters (named EP25–EP30), operating under continuous mode sonication (i.e., 100% DC), were also administered with three sonication durations (60, 100, and 140 ms) and two levels of *in situ* spatial-peak pulse-average intensity (I_sppa_) of 15.8 and 18.2 W/cm^2^. These three sonication durations in continuous mode sonication were chosen in order to deliver the same level of acoustic energy as those used in pulsed mode sonication (EP1–EP24). The sonication in stimulation trials was given every 5 s (i.e., ISI = 5 s) to prevent potential heating of the tissue (please see **‘Estimation of potential thermal effects from FUS’** below).

To evaluate the suppressive FUS parameters, eight parameter sets of varying tone-burst durations (0.5 and 1.0 ms), DCs (3 and 5%), and levels of *in situ* I_sppa_ (5.4 and 11.6 W/cm^2^) were examined (named SP1–SP8, see Table 2). Each suppressive sonication set was given for a duration of 2 min once per session (therefore, ISI did not apply). Due to this relatively long sonication duration (compared to 200 ms for the excitatory FUS), continuous mode sonication was not tested to avoid potential heating of the tissue.

To estimate the *in situ* intensity of the administered ultrasound, we retrospectively measured the acoustic pressure attenuation through coupling hydrogels and extracted sheep skulls (n = 6) using a hydrophone (HNC200, Onda) in a degassed water tank. The levels of attenuation in terms of intensity were 16.3 ± 0.3% (i.e., 83.7% transmission) and 69.4 ± 0.3% (i.e., 30.6% transmission) through the coupling cryogel and the extracted sheep skull, respectively. Based on these findings, the *in situ* I_sppa_ (W/cm^2^) was estimated using an intensity transmission level of 25.6% (i.e., 30.6% × 83.7% transmission with respect to the level calibrated in the degassed water). The potential attenuation of tissues such as scalp and dura were not accounted for in this estimation.

### Electrophysiological assessment of the bimodal effects of FUS

To examine the efferent effects of excitatory FUS, EMG activity from both hind limbs (i.e., contralateral and ipsilateral EMG) was measured using a dual-channel data acquisition system (BioAmp ML408 with PowerLab 4/35, ADInstruments, Colorado Springs, CO) while monitoring the presence of overt muscle twitches or leg movement during sonication. Subdermal wire electrodes (SWE-L-25, Ives EEG Solutions, Newburyport, MA) with a 1.5 mm silver chloride (AgCl) tip were inserted subcutaneously over the gastrocnemius muscle with a ~3 cm distance between the positive and negative electrodes. A reference cup electrode was placed on the skin between the hooves. The wool over both upper hind limbs and between the hooves was trimmed to expose the skin for placement of electrodes.

Because the amplitude and temporal features (i.e., frequency spectrum and shape) of the EMG signals may vary depending on the electrode configurations (e.g., shape and type of electrode) [66–68] and the data acquisition hardware settings, we first measured the EMG response from mechanical stimulation of the muscle over the peroneal/tibial nerve of the hind leg (from ‘SH4’), which showed a biphasic shape instead of a repetitive EMG firing pattern (Supporting Information S1A and S1B Fig show an example of the measured EMG). Passive muscle actuation is known to generate a comparable EMG signal from the active limb motion in humans [69, 70].

Then, the time-locked EMG signal was acquired from 0.5 s before to 1 s after the onset of each FUS sonication event (10 kHz sampling rate, low-pass filter of 30 Hz high cut-off; LabChart 7, ADInstruments). Twenty excitatory FUS stimulations were given in each session. Multiple sonication sessions (up to four) were administered to each animal (see Supporting Information S1–S4 Tables for more detail; the number of stimulations, therefore, ranges from 20–80). As the EMG signal can be confounded by spontaneous muscle activity or respiratory motion, we used the following criteria to detect the FUS-related signals: (1) magnitude (difference between the distinct positive and negative peaks) over 1.5 μV, and (2) time interval between FUS onset to the emergence of the first negative peak (defined as the ‘latency’ herein) in the range of 0–250 ms, considering the sonication duration of 200 ms and the sheep’s nerve conduction velocity of ~100 m/s [71]. Here, a magnitude of 1.5 μV was chosen based on averaged one standard deviation (1.48 ± 0.92 μV, n = 10 sheep) of the EMG signal fluctuations measured during 1 min of resting-state. The assessment window of 0–250 ms for analyzing the latency of EMG responses was based on the presence of a broad onset latency distribution of response elicited by FUS brain stimulation of the motor cortical area in previous studies [28, 31, 72, 73]. We excluded from further analysis any peaks (1) with signal magnitude over 20 μV (due to the possibility of signal artifact) or (2) with a time gap greater than 200 ms between the positive and negative peaks (related to motion artifact). The response rate for each sonication parameter per animal was calculated as a ratio of the number of elicited EMG responses to the total number of FUS stimulations. Considering the possible presence of reflex-type startle responses among acquired data [74, 75], EMG responses with short latency (< 25 ms) were not included as successful excitatory responses for the response rate calculation.

To assess the suppressive effects, the EEG SEP induced by unilateral electrical stimulation of the right hind leg muscle was monitored. EEG data was acquired from two subdermal EEG electrodes (SWE-L-25, Ives EEG Solutions) inserted (1) under the skin over the left rostral portion of the skull and (2) ~2 cm left of the bregma (based on MRI images), using the same dual-channel data acquisition system (BioAmp ML408 with PowerLab 4/35, ADInstruments). Reference electrodes were subcutaneously applied over the left posterior region of the occipital bone, and a ground cup electrode was placed between the hooves of the right fore limb. To elicit the SEP, electrical stimulation (10–15 mA electrical currents, duration of 50 μs, and frequency of 2 Hz) was given to the gastrocnemius of the right hind limb using a surface stimulator (MLADDF30, ADInstruments). The corresponding EEG was recorded from 50 ms before to 100 ms after the onset of electrical stimulation (10 kHz sampling rate, band-pass filter at 0.5–200 Hz; LabChart 7, ADInstruments). The time-locked EEG signal was measured every 0.5 s a total of 120 times (thus, 1 min for each SEP acquisition) and averaged to represent SEP.

The SEP was measured three times (labeled B1–B3) to establish the baseline condition before applying sonication. Subsequently, FUS was delivered to the targeted brain areas for 2 min while measuring two sets of SEPs (labeled F1 and F2). After the end of sonication, five additional sets of SEP (labeled P1–P5) were acquired in succession. An example of the obtained SEP signal is displayed in Supporting Information S1C Fig. To provide active control conditions, we also delivered sonication to the S1 and thalamic area in the right hemisphere across five sheep, using the sonication parameters of SP3 and SP7 (a total of 17 sets of measurement were taken). The distance between the target and the corresponding control sites was 18.8 ± 5.2 mm (n = 5) for the S1 and 14.5 ± 4.7 mm (n = 5) for the thalamic area.

To examine the degree of suppression per animal, we measured the magnitude between the negative peak at ~40 ms (N_40_) and the positive peak at ~50 ms (P_50_) of the SEP (i.e., P50 –N_40_ in Supporting Information S1C Fig) after the onset of electrical stimulation. The SEP magnitude from acquisition sets (i.e., B1–B3, F1, F2, and P1–P5) was normalized with respect to the averaged magnitude across pre-sonication baseline conditions (i.e., B1–B3), whereby the averaged value acquired from the baseline conditions was set to zero.

### Statistical analysis

Data were presented as the mean ± standard deviation (unless otherwise noted), and statistical analyses were performed using Matlab (Mathworks, Natick, MA). First, grand mean response rates between the contralateral and ipsilateral EMGs elicited by excitatory FUS were compared using a one-tailed *t*-test with a confidence level of 99% (p < 0.01), for all 30 different parameter sets across eight animals, for stimulation of M1 and thalamus. Further comparisons, in terms of response rate, were made across different DCs, tone-burst durations, and I_a_ (I_sppa_), by primarily analyzing the response rates of EMGs acquired contralateral to FUS stimulation. One-way analysis of variance (ANOVA) followed by *post-hoc* least significance difference (LSD) test was performed for the comparisons across the different DCs and tone-burst durations. For the comparison between two different I_a_s, a paired two-tailed *t*-test was performed with a confidence level of 95% (p < 0.05).

Based on these parametric analyses (see Results), the time-locked averaged EMG traces contralateral to FUS stimulation given at 70% DC (i.e., EP17–EP24) were compared to those measured in the absence of sonication (no FUS condition), in terms of mean EMG amplitude at each time point (0.1-ms stepwise) using a one-tailed *t*-test with a confidence level of 99% (p < 0.01) for both M1 and thalamic excitations. Due to the distributed peak latencies of the elicited EMG responses, the time-locked EMG signals were averaged and analyzed separately for every 25-ms latency bin in a range of 25–250 ms.

In the case of SEP data from suppressive sonication sessions, one-way ANOVA with *post-hoc* Tukey-Kramer analysis was first used to examine whether or not the control condition’s SEP magnitudes changed significantly across the acquisition sets (i.e., B1–B3, F1, F2, and P1–P5). Then, *t*-test (one-tailed) with a confidence level of 99% (p < 0.01) was used to compare the group-averaged normalized SEP peak magnitudes between the control condition and sonication sessions across acquisition set (B1–P5). Based on these analyses (see Results), group-averaged SEP traces acquired from the use of SP1 and SP3 parameter conditions for each data acquisition segment (B1–P5) were compared to that of the control condition, in terms of mean EEG amplitude at each time point (0.1-ms stepwise), using a one-tailed *t*-test with a confidence level of 99% (p < 0.01) for both S1 and thalamic suppressions.

### Estimation of potential thermal effects from FUS

To examine the potential thermal effects from sonication, we estimated the temperature increase at the focus by sequentially solving the Khokhlov-Zabolotskaya-Kuznetsov (KZK) equation and bio-heat transfer equation through an open source high intensity FUS (HIFU) simulator [76]. The simulation resolution was set to 0.5 mm based on the previous numerical study of FUS propagation through the skull [47]. The sonication parameters of EP30 and SP6 were assessed for the simulation because they would yield the highest energy deposition among the excitation and suppression sonication parameters, respectively. The simulation was conducted with a temporal resolution of 0.2 ms using the acoustic properties (speed of sound of 1550 m/s, density of 1045 kg/m^3^, attenuation coefficient of 80 dB/m/MHz) and thermal properties of the brain (specific heat of 3696 J/kg/K, thermal conductivity of 0.55 W/K/m, perfusion rate of 14.1 kg/m^3^/s) [32].

We also measured the temperature change from sonication of a tissue phantom (Zerdine, CIRS Inc., Norfolk, VA) using the experimental conditions and sonication parameters of EP30 and SP6. The phantom was placed above a 2 cm-thick acoustic gel (Aquaflex, Parker Laboratories), which was placed over a 5 mm-thick rubber pad. The coupling hydrogel cone, phantom, and acoustic gel were heated to 36–39 °C prior to sonication using a thermal pad (TP650, Gaymar Industries Inc., Orchard Park, NY). An infrared thermal camera (with a sensing sensitivity of ~0.5 °C; C3, FLIR Systems Inc., Wilsonville, OR) was used to measure the temperature of the phantom at the focus five times with an interval of ~5 min between measurements.

### Post-sonication EEG, MRI, behavioral monitoring, and histology

Baseline resting-state EEGs after the application of FUS were acquired for 3 min from four sheep to assess for any gross abnormalities in electrographic neural activity, such as presentation of repetitive EEG spikes with voltage values that exceed 5 μV that are suggestive of epilepsy. Resting-state EEGs were also acquired in two animals before the application of FUS. Seven sheep underwent MRI immediately after additional FUS sessions (using sonication parameters of SP1 and EP18) without evaluation of physiological responses, in order to assess the presence of gross tissue damage or disruption of the blood-brain barrier (BBB). T_1_-weighted FSE images (field of view 18 × 18 cm^2^, slice thickness 3 mm, image matrix 256 × 256, TR/TE = 500/13 ms, echo train length 4, flip angle 90°) of the brain were acquired before and after injection of an MR contrast agent (Magnevist, Bayer, Wayne, NJ) at a dose of 0.2 mL/kg.

Post-sonication behavior of all sheep was monitored regularly (every one to three days) to check for the presence of any abnormalities throughout the survival period. The sheep were euthanized after their last sonication session at varying time points (n = 3 for acute, n = 2 for 1 week, n = 3 for 2 weeks, n = 2 for 2 months), and their brains were extracted and divided into the left and right hemispheres. Both hemispheres were sliced into 5 sections in the caudal to rostral direction so that the location of the middle three sections, each ~10 mm thick, included the sonication targets. The samples were fixed in 10% buffered formalin phosphate for three days, and were further cut in half (~5 mm) to include the slice containing the sonication targets (the M1/S1 and thalamus) as guided by the high-resolution T_1_-weighted MRI. The corresponding slice was divided into ~25 × 20 × 5 mm^3^ segments in the superior/inferior orientation to fit into the histological sample cassettes, and these segments were fixed in formalin for one week. The brain tissue blocks were paraffin-embedded, and 7 μm-thick microtome sections (5–8 slices) were sampled from different parts of the block for histological staining. Control tissue segments were also prepared from the unsonicated M1/S1/thalamus (n = 4 animals) in the right hemisphere and underwent the same sectioning process.

For the histological analysis, hematoxylin and eosin (H&E; GHS-2-16, Sigma-Aldrich, St. Louis, MO) staining, vanadium acid fuchsin (VAF)-toluidine blue staining (A3908, Sigma-Aldrich), and immunohistochemistry (IHC) of glial fibrillary acidic protein (GFAP; ab7260, Abcam, Cambridge, UK) staining and caspase-3 (ab4051, Abcam) staining were performed. Leica Biosystems Refine Detection Kit (DS9800, Leica Biosystems, Buffalo Grove, IL) with citrate antigen retrieval with a primary antibody dilution factor of 1:300 for GFAP and 1:500 for caspase-3 was utilized for IHC. The histology slides were imaged using an automated microscope cell imaging system (EVOS FL Auto 2, Thermo Fisher Scientific, Waltham, MA). We examined H&E for necrosis, VAF for ischemic neurons, GFAP for glial infiltration and degeneration of neurons, and caspase-3 for apoptotic cells, to detect any signs of tissue damage.

The entire timeline of the experimental procedures, from neuroimaging acquisition to sacrifice, across the animals is depicted in Supporting Information S2 Fig.

## Results

### Effects of sonication parameters in excitatory FUS

#### Comparison of response rates between contralateral and ipsilateral EMG

**Fig 2:**
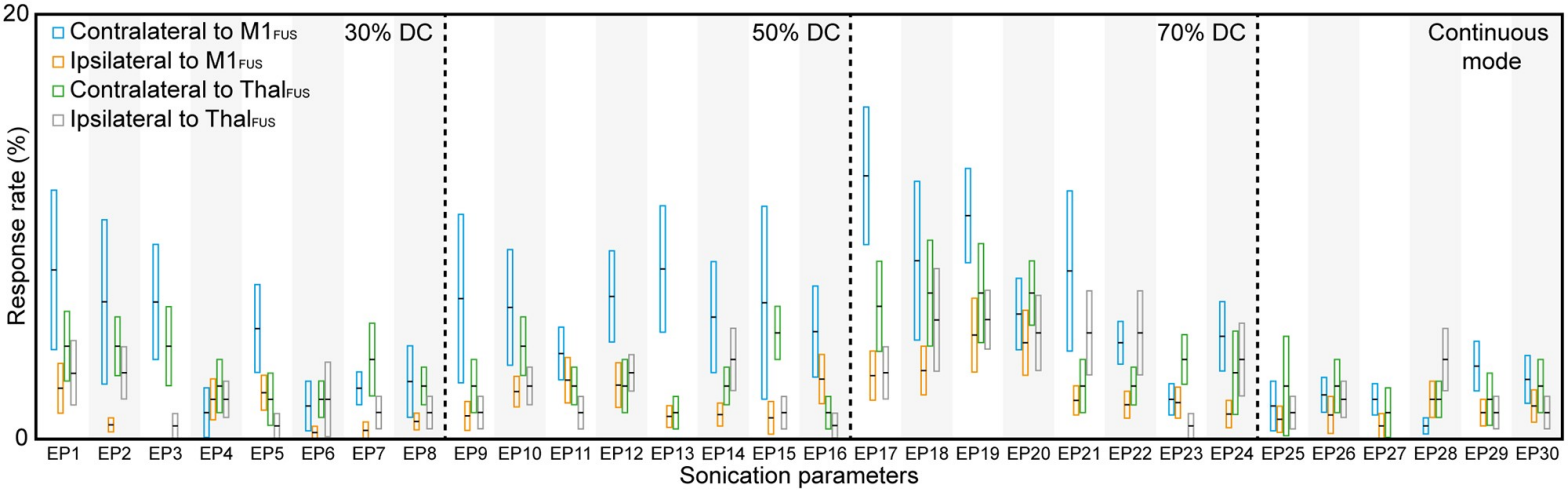
Results of excitatory sonication to the M1 and thalamus (Thal). The group-averaged EMG response rates (with standard error bars) measured from bilateral hind limbs for sonication parameters EP1–EP30 (see Table 1), evaluated across ten sheep (8800 sonication trials for the M1 stimulation and 4800 trials for the thalamic stimulation). The box indicates the range of positive and negative standard error, and the black horizontal line in the box indicates the average value.

Fig 2 shows the group-averaged response rates measured from both hind legs across the tested excitatory sonication parameters (EP1–EP30: the detailed animal-specific data is given in the Supporting Information S1–S4 Tables). Variations exist in the response rates across the parameters depending on the location of sonication and the side of EMG measurement (whether it is ipsilateral or contralateral to the stimulation), with an averaged maximum response rate of 12.4 ± 9.2% (n = 8) (measured from the hind leg contralateral to the sonication) when EP17 (0.5 ms tone-burst duration, 70% DC, and 15.8 W/cm^2^ I_sppa_) was used to stimulate the M1 in the left hemisphere. None of the animals showed any visible movement of the hind legs throughout the procedure.

The response rates obtained from EMG data from both legs were assessed and compared to each other. In M1 stimulation, the response rate from the right hind leg contralateral to stimulation (4.9 ± 7.3%; n = 240; 30 parameter sets across eight animals) was higher than that measured from the ipsilateral (left) hind leg (1.7 ± 2.7%; n = 240; one-tailed *t*-test, *t*-value = 6.93, degrees of freedom = 239, p = 1.91 × 10^−11^). In thalamic stimulation, the response rate was lower (maximum response rate of 6.9%) than that from contralateral M1 stimulation (n = 240; one-tailed *t*-test, *t*-value = 6.93, degrees of freedom = 239, p = 0.0009) and was highest when EP18, EP19, and EP20 were used. Similar to the findings from the M1 stimulation, a higher EMG response rate was observed from the right hind leg (3.3 ± 4.4%; n = 240) compared to the left hind leg across sonication parameters (2.4 ± 3.8%; n = 240; one-tailed *t*-test; *t*-value = 2.78, degrees of freedom = 239, p = 0.0029).

#### Comparison across different duty cycle conditions

**Fig 3:**
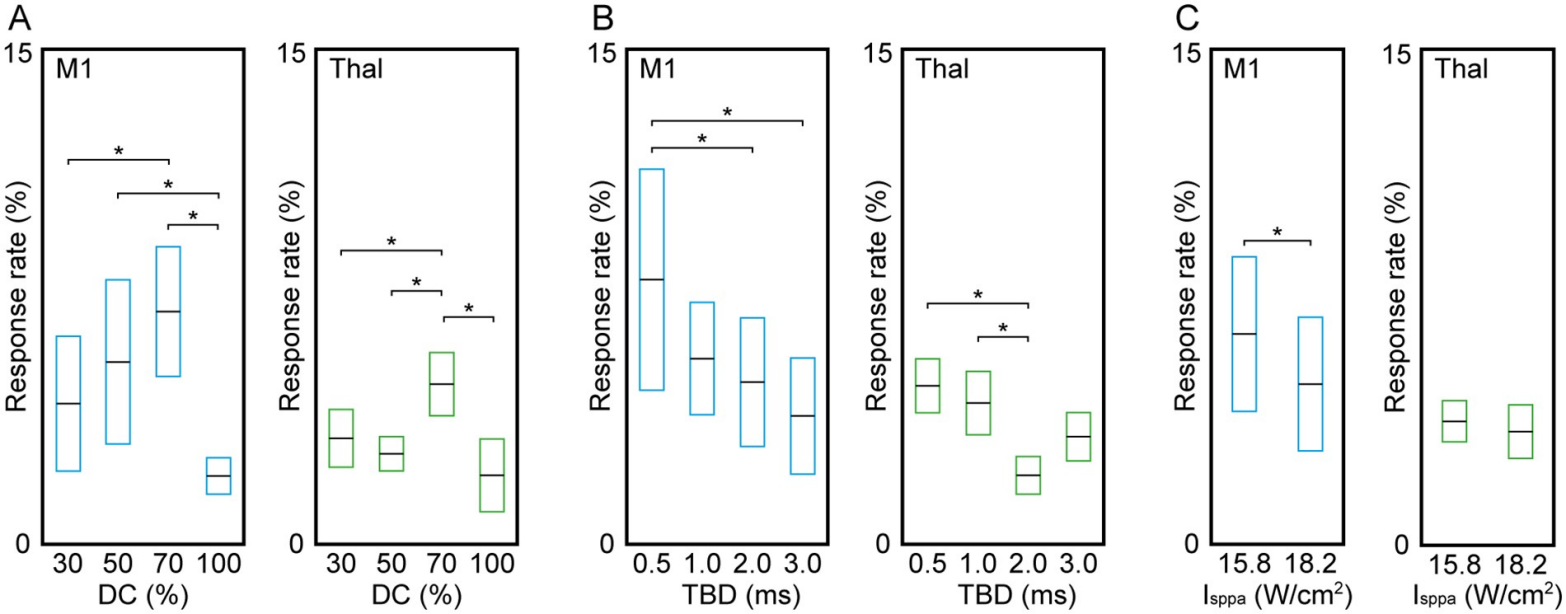
The response rate in excitatory sonication to the M1 and thalamus, comparing different DCs, tone-burst durations (TBDs), and I_sppa_. Averaged response rate (horizontal black line in box) with standard error (box) in contralateral EMG was plotted across the different sonication parameters in terms of (A) DC, (B) TBD, and (C) I_sppa_. The bracket with the asterisk indicates significant differences (one-way ANOVA with LSD *post-hoc* analysis in panels A and B, *post-hoc* test p < 0.05, and paired two-tailed *t*-test in panel C, p < 0.05) of the response rate between different sonication parameters.

In M1 stimulation, significant differences across the four different DCs (30, 50, 70 and 100%) were analyzed (one-way ANOVA, *F* = 4.8, p = 0.0029, followed by LSD *post-hoc* analysis). The use of 70% DC (7.0 ± 5.5%; n = 64; 8 parameters across eight animals) yielded the highest response rate with significant differences (*post-hoc* test p < 0.05, Fig 3A) when compared to the use of 30% (4.3 ± 5.8%; n = 64) and 100% DCs (2.1 ± 1.6%; n = 48; 6 parameters across eight animals). The use of continuous sonication (i.e., 100% DC) showed the lowest response rate with significant differences (*post-hoc* test p < 0.05, Fig 3A) when compared to those from 50% DC (5.5 ± 7.0%; n = 64) or 70% DC, while the difference was marginal with the use of 30% DC (*post-hoc* test p = 0.1091). Similar to the observations from M1 stimulation, one-way ANOVA (*F* = 4.32, p = 0.0055) with LSD *post-hoc* analysis of the response rate from thalamic stimulation showed that the use of 70% DC (4.8 ± 2.7%; n = 64) resulted in a significantly higher response rate (*post-hoc* test p < 0.05) than the use of 30% (3.2 ± 2.5%; n = 64), 50% (2.7 ± 1.5%; n = 64), and 100% (2.1 ± 3.1%; n = 48) DCs. The continuous sonication (i.e., 100% DC) showed the lowest response rate. These results indicate that the use of 70% DC offers superior stimulation efficiency compared to the other DCs used in the present study.

#### Comparison across different tone-burst duration conditions

Tone-burst durations of 0.5, 1, 2, and 3 ms also showed different response rates in both M1 and thalamic stimulation (one-way ANOVA with LSD *post-hoc* analysis). In M1 stimulation, although statistically marginal (one-way ANOVA, *F* = 2.44, p = 0.0661), the use of 0.5 ms tone-burst duration (8.0 ± 9.5%; n = 48; 6 parameters across eight animals) resulted in the highest response rate (*post-hoc* test p < 0.05, Fig 3B) when compared to the use of 2 ms (4.9 ± 5.5%; n = 48) or 3 ms tone-burst durations (3.9 ± 5.0%; n = 48). In thalamic stimulation, the use of 0.5 ms (4.8 ± 2.3%; n = 48) or 1 ms tone-burst durations (4.3 ± 2.7%; n = 48) yielded higher response rates, with significant differences (one-way ANOVA, *F* = 3.59, p = 0.0148; *post-hoc* test p < 0.05) when compared to the use of 2 ms tone-burst duration (2.1 ± 1.6%; n = 48).

#### Comparison between I_a_ conditions

In M1 stimulation, the use of 15.8 W/cm^2^ I_sppa_ generated a higher response rate (6.4 ± 6.6%; n = 96; 12 parameters across eight animals) than the use of 18.2 W/cm^2^ (4.8 ± 5.7%; n = 96) with statistical significance (paired two-tailed *t*-test, p < 0.05, Fig 3C). A similar trend was observed with thalamic stimulation, in which the application of 15.8 W/cm^2^ I_sppa_ showed a slightly higher response rate (3.8 ± 1.8%; n = 96) compared to the use of higher I_sppa_ (18.2 W/cm^2^, 3.4 ± 2.3%, n = 96), but without statistical significance (p = 0.712).

#### Latency of responses

**Fig 4:**
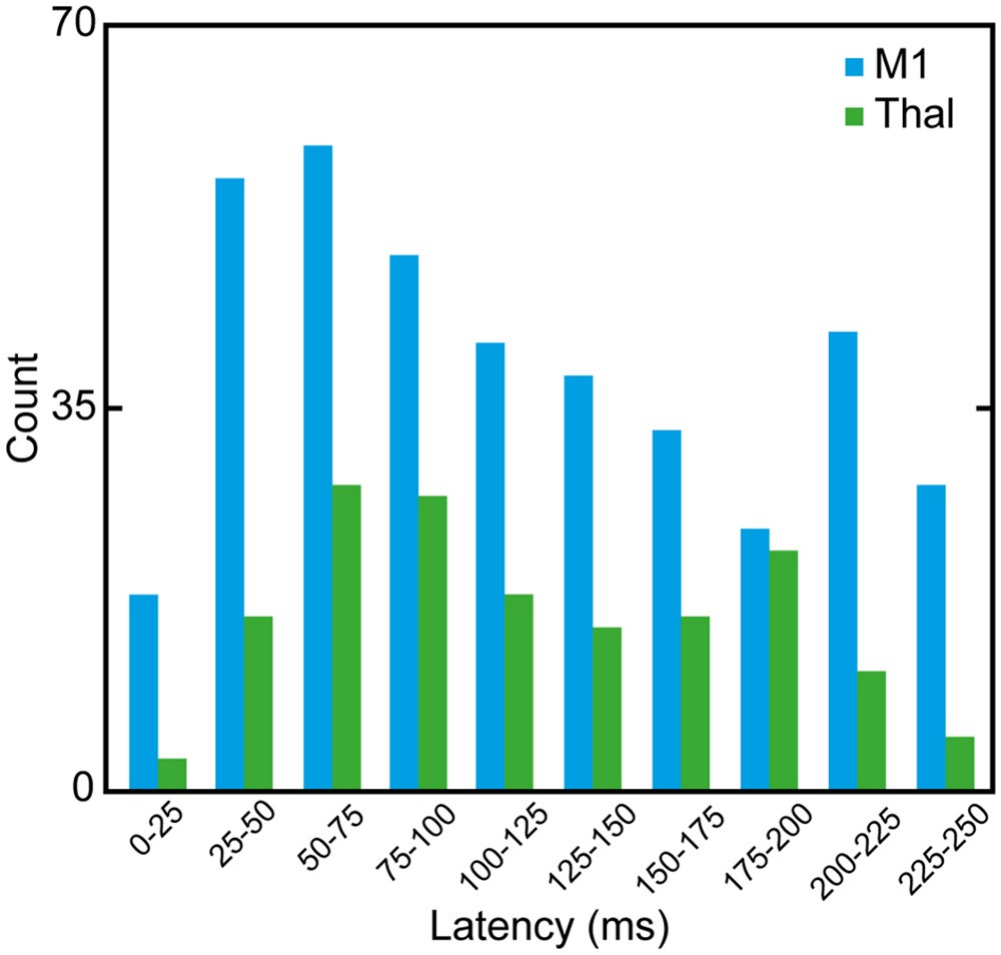
Histogram of onset latencies of contralateral hind limb EMG signals after FUS onset. Blue bars indicate the responses from M1 stimulation sessions while green bars indicate those from thalamic stimulation sessions.

A histogram of the latency of EMG signal peak from FUS onset (i.e., time to first negative peak, see Supporting Information S1B Fig) was constructed across all sonication parameters (Fig 4), and EMG data from all successful excitatory responses contralateral to FUS stimulation were grouped (370 for M1 stimulation and 158 for thalamic stimulation). Considering the potential inclusion of reflex-type startle responses among the short latency EMG responses [74, 75], data with latencies shorter than 25 ms were not included as successful excitatory responses and were excluded in statistical analysis in Figs 2 and 3 (see Methods). Latency values ranging from 50–75 ms were most frequently observed from both M1 and thalamic stimulations. The averaged latency of EMG responses was 116.0 ± 13.9 ms for M1 stimulation and 119.8 ± 10.3 ms for thalamic stimulation.

#### Time-locked averaged EMG in excitatory sonication

**Fig 5:**
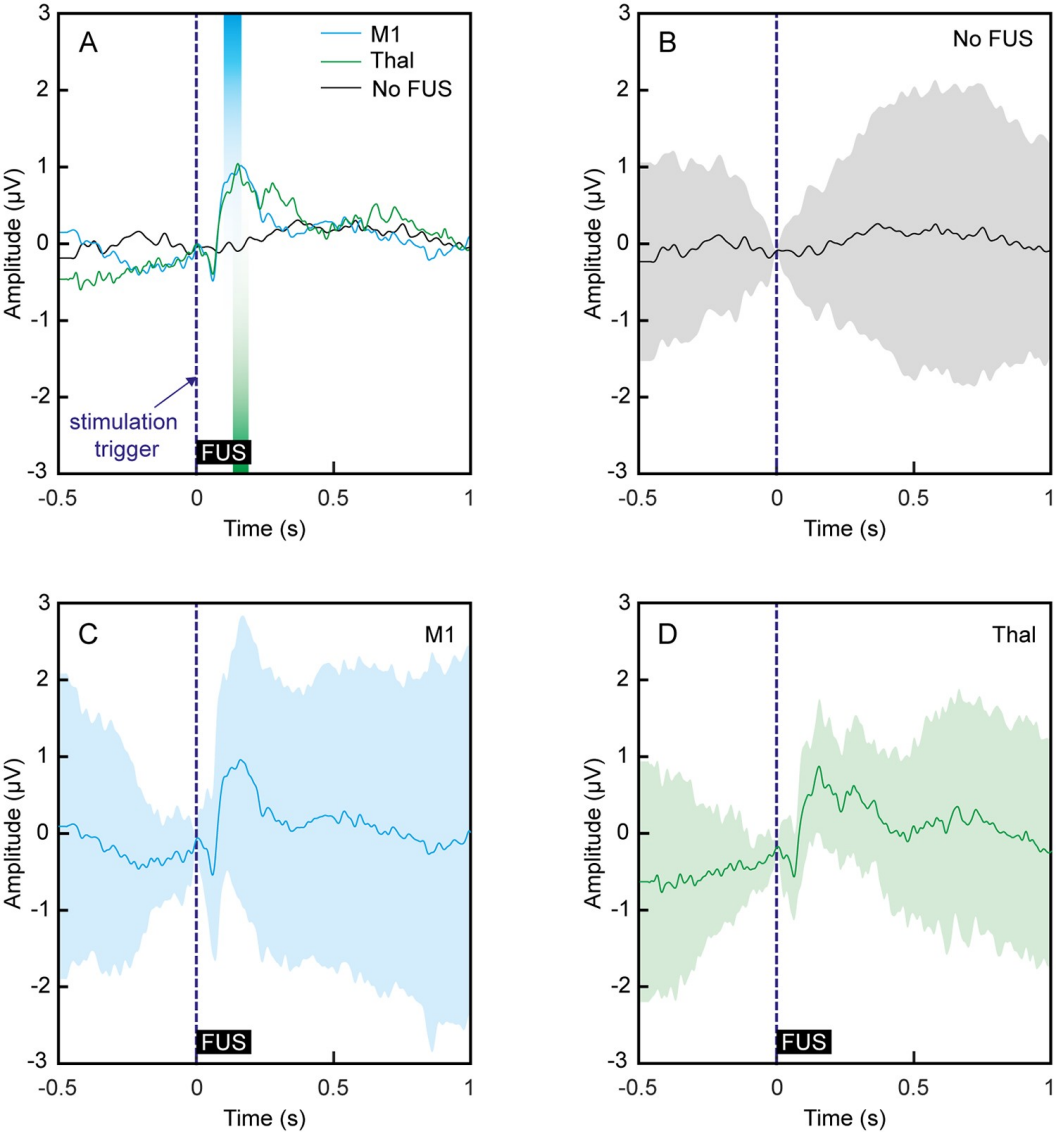
Time-locked contralateral EMG measured from M1/thalamic stimulation. The EMG signals were averaged, using the results obtained from the use of 70% DC (i.e., EP17–EP24) having an onset latency between 50 and 75 ms. (A) The averaged data from stimulation of the M1 and thalamus is displayed in the blue and green lines, respectively. The data obtained in the absence of sonication is plotted in the black line (labeled as ‘No FUS’). The baseline signal drift/offset was removed from all individual EMG data with respect to FUS onset. The colored bars indicate regions of significant differences (p < 0.01, one-tailed *t*-test) in the amplitude obtained from M1 (in blue) and thalamic (in green) stimulation compared to the amplitude obtained when FUS was not given (i.e., ‘No FUS’). Dashed lines indicate the onset timing of FUS sonication, and thick solid black bars represent the duration of sonication. (B–D) Each of the averaged signal traces shown in (A) is separately shown for (B) No FUS, (C) M1 and (D) thalamic stimulation, respectively. The shaded envelopes indicate the standard deviation of the averaged baseline/EMG signal traces. The zero padded temporal features of the signal were due to the application of low-pass digital filter (30 Hz).

The time-averaged EMG responses contralateral to FUS stimulation given at 70% DC (i.e., EP17–EP24) were examined as a representative example (Fig 5). Due to the distributed peak latencies of the elicited EMG responses (see Fig 4), only EMG signals with a latency of 50–75 ms were shown. The EMG signals of other latency bins are displayed in Supporting Information S3 Fig. The time-locked averaged EMG signals from stimulation of the M1 (n = 24, blue line) and thalamus (n = 13, green line) were compared to those measured in the absence of sonication (n = 20, black line), and the duration during which there is a significant difference in amplitude (one-tailed *t*-test, p < 0.01) is marked by the blue and green bars, respectively. EMG signals measured from both the M1 and thalamus contralateral to sonication showed distinctive peaks in the range of 100–190 ms.

### Effects of sonication parameters in suppressive FUS

**Fig 6:**
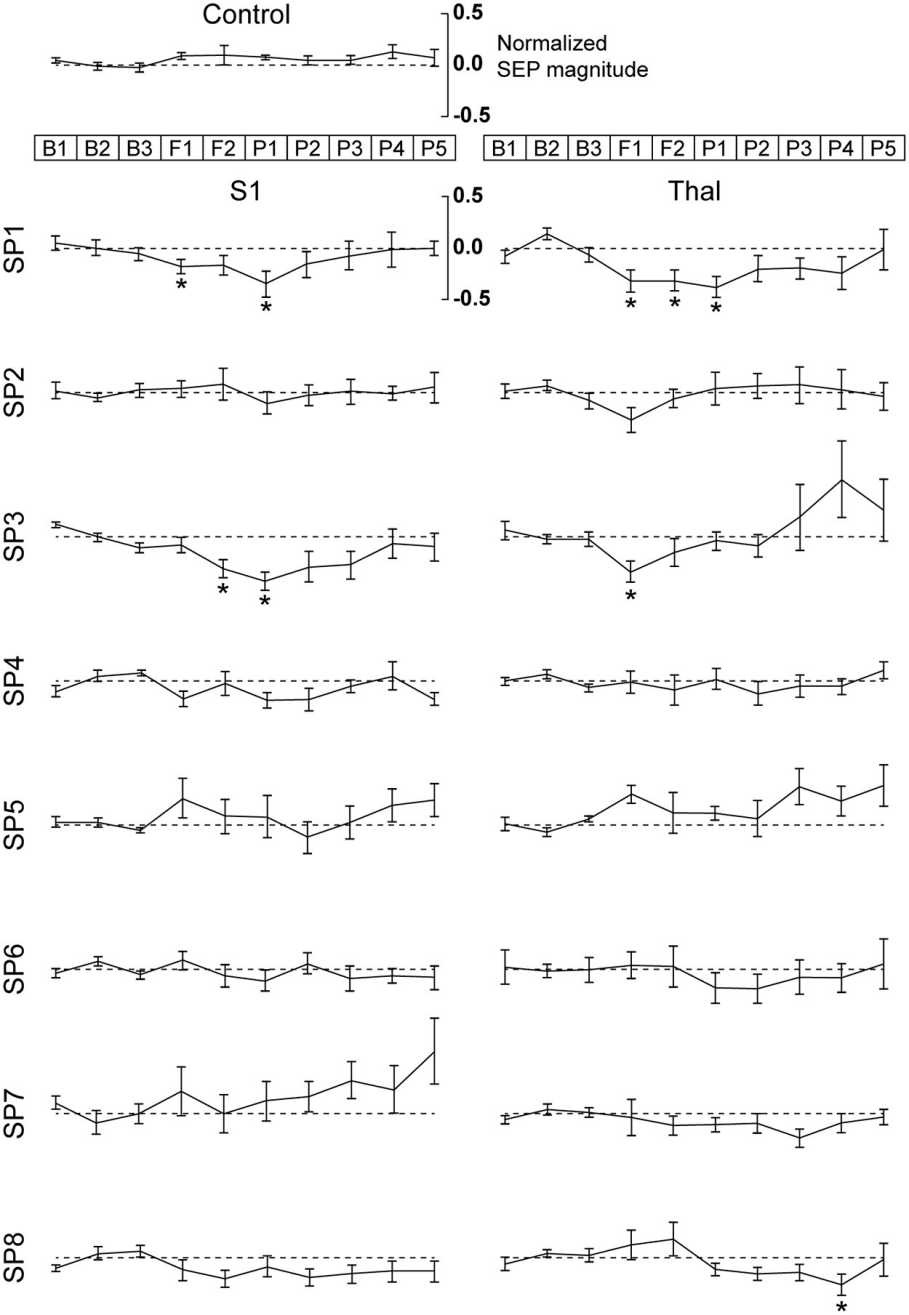
Normalized SEP magnitude (P_50_ –N_40_; averaged across the animals shown with standard error) before (B1–B3), during (F1, F2), and after (P1–P5) sonication of the contralateral S1 (left column) and thalamus (right column). The top shows the data acquired from the control condition (i.e., sonication of the ipsilateral S1 and thalamus), with each row representing the data acquired with each sonication parameter set (SP1–SP8; refer to Table 2 for detailed parameters). The dashed line indicates the normalized signal level averaged across baseline B1–B3 segments (set to zero). The asterisks indicate statistically significant differences (one-tailed *t*-test, p < 0.01) in SEP magnitude when compared to the control condition.

Across the different suppressive sonication parameters (SP1–SP8), the group-averaged time-progression (from B1 to P5) of normalized SEP peak magnitude (P_50_ –N_40_) observed from the sonication targets (i.e., the S1 and thalamus) is shown in Fig 6.

During the control condition (i.e., sonication of the ipsilateral S1/thalamus), the SEP magnitude did not change over time (across 10 acquisition sets, one-way ANOVA with *post-hoc* Tukey-Kramer analysis; n = 17, degrees of freedom = 9, *F*-value = 0.6542, all p > 0.1), suggesting that sonication of the ipsilateral sensory circuits did not affect the SEP from the contralateral side. When SEP magnitudes from different sonication parameters were compared to those measured from the control condition across time, the use of SP1 and SP3 showed significant reduction of SEP magnitude during S1 sonication (i.e., F1 or F2, one-tailed *t*-test, p < 0.01, marked as ‘*’ in Fig 6). The degree of SEP magnitude reduction with the use of SP1 and SP3 was 17.8 ± 24.1% (F1) and 31.4 ± 28.2% (F2), respectively. A similar degree of reduction in SEP magnitude (one-tailed *t*-test, p < 0.01, marked as ‘*’ in Fig 6) was also observed during thalamic sonication using SP1 and SP3 (ranging from 31.4 ± 28.3% to 34.3 ± 27.9% reduction in F1 and/or F2 segments). The reduced SEP magnitude persisted during the post-sonication period of P1 with the use of SP1 (with both S1 and thalamic sonication) and SP3 (with S1 sonication). The use of other parameters sets (i.e., SP2, SP4–SP8) did not yield significant differences in SEP magnitude compared to those obtained from the control condition during and after sonication periods, with the exception of a depressed SEP magnitude in the P4 segment when the SP8 parameter set was used to sonicate the thalamus. These results suggest that the use of SP1 and SP3 (i.e., tone-burst duration of 0.5 ms and I_sppa_ of 5.4 W/cm^2^; acquired at 3 and 5% DCs) temporarily suppressed the SEP.

#### Examination of SEP traces

**Fig 7:**
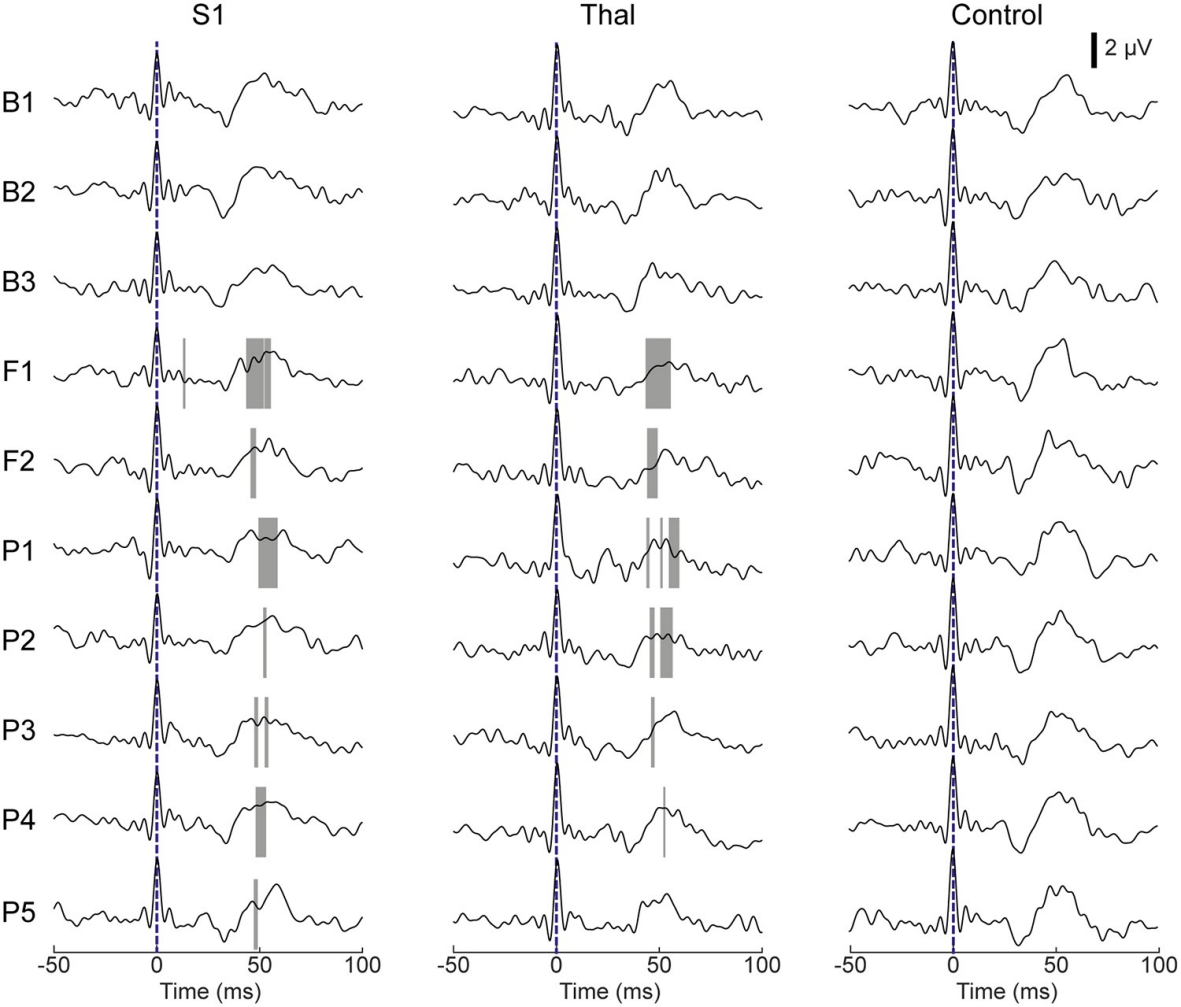
Group-averaged SEP (acquired from the use of SP1 and SP3 parameter conditions) across data acquisition segments for the sonication targets (S1, thalamus). The time-locked averaged SEPs are shown before (B1–B3), during (F1, F2), and after (P1–P5) sonication of the S1 (left column, n = 17 across 9 sheep) and thalamus (middle column, n = 16 across 8 sheep). The SEPs acquired from the control condition (i.e., sonication of the ipsilateral S1/thalamic targets, right column, n = 17 across 5 sheep) are also shown. Gray bars in the plot indicate the time intervals that showed significant differences in amplitude compared to the control condition (one-tailed *t*-test, p < 0.01).

The SEP data acquired from the FUS sessions that showed effective suppression (i.e., using SP1 and SP3) was grouped and compared to the corresponding control condition in a time-locked fashion. The time portions that showed significant reductions in EEG SEP amplitude (one-tailed *t*-test, p < 0.01) are marked with gray bars (Fig 7).

Across baseline time segments (B1–B3), the measured SEPs were not statistically different from those acquired during the control condition for sonication of the S1 and thalamus. Upon administration of FUS, SEP amplitude was reduced in the 40–60 ms interval, which persisted during the post-sonication observation period. It is notable that the time portion of the SEP showing signal reduction became narrower during the progression of the post-FUS period, whereby the SEP obtained from thalamic stimulation became indistinguishable from the corresponding control condition by the P5 segment.

### Estimation of potential thermal effects

**Fig 8:**
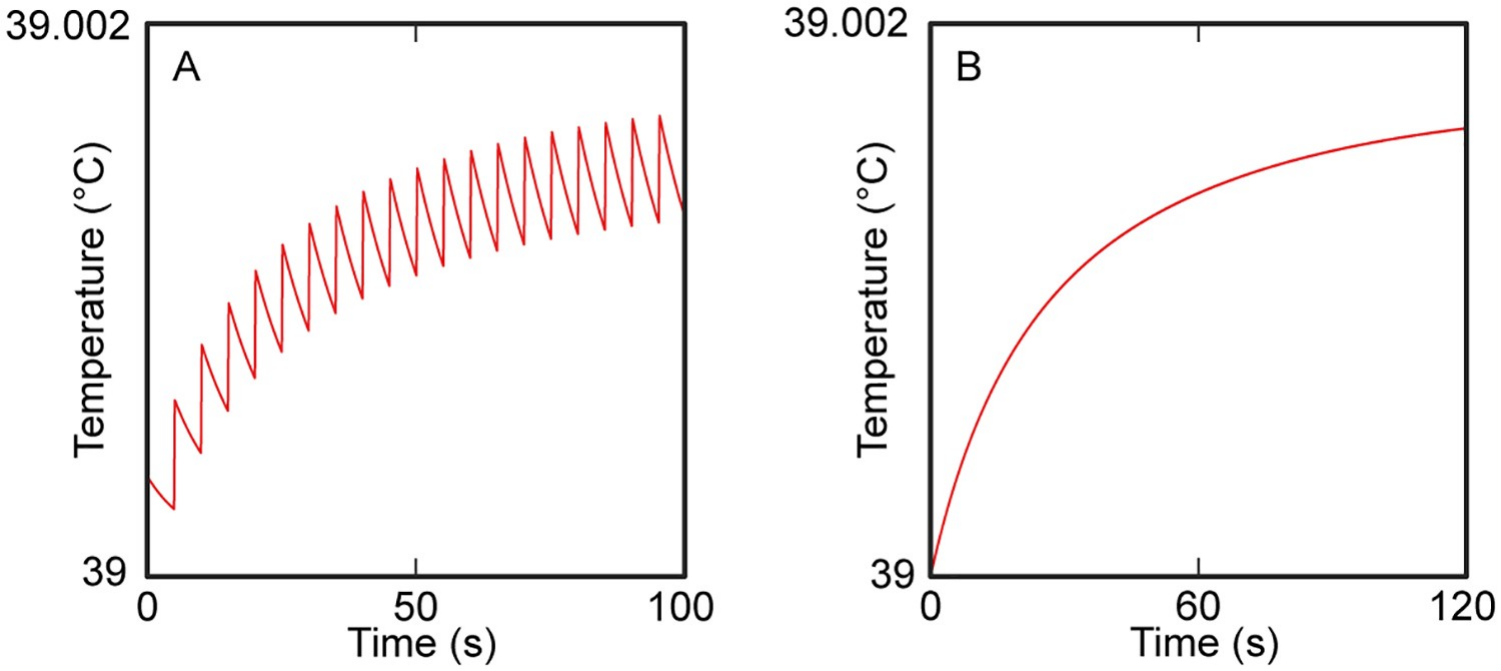
Estimated temperature rise at the focus. (A) 20 sonication repetitions with 5 s ISI using excitatory parameter set of EP30. (B) Sonication duration of 2 min using suppressive parameter set of SP6. The trace of thermal change was illustrated with the raw data of numerical simulation performed with a temporal resolution of 0.2 ms.

Fig 8 displays the results of the thermal analysis using the sonication parameters of EP30 and SP6. In the excitatory sonication condition (EP30), the temperature increase at the acoustic focus was estimated as 0.0017 °C through 20 repetitions of sonication every 5 s (i.e., ISI) with a 140 ms sonication duration. In the suppressive sonication condition (SP6), the temperature increase at the acoustic focus was estimated as 0.0016 °C after a 2 min sonication. The temperature change after sonication of a tissue phantom was measured as -0.12 ± 0.18 °C (n = 5) for suppressive sonication and 0.12 ± 0.04 °C (n = 5) for excitatory sonication, both of which were under the detection sensitivity of infrared thermometry.

### Post-sonication behavior monitoring and histological assessment

**Fig 9:**
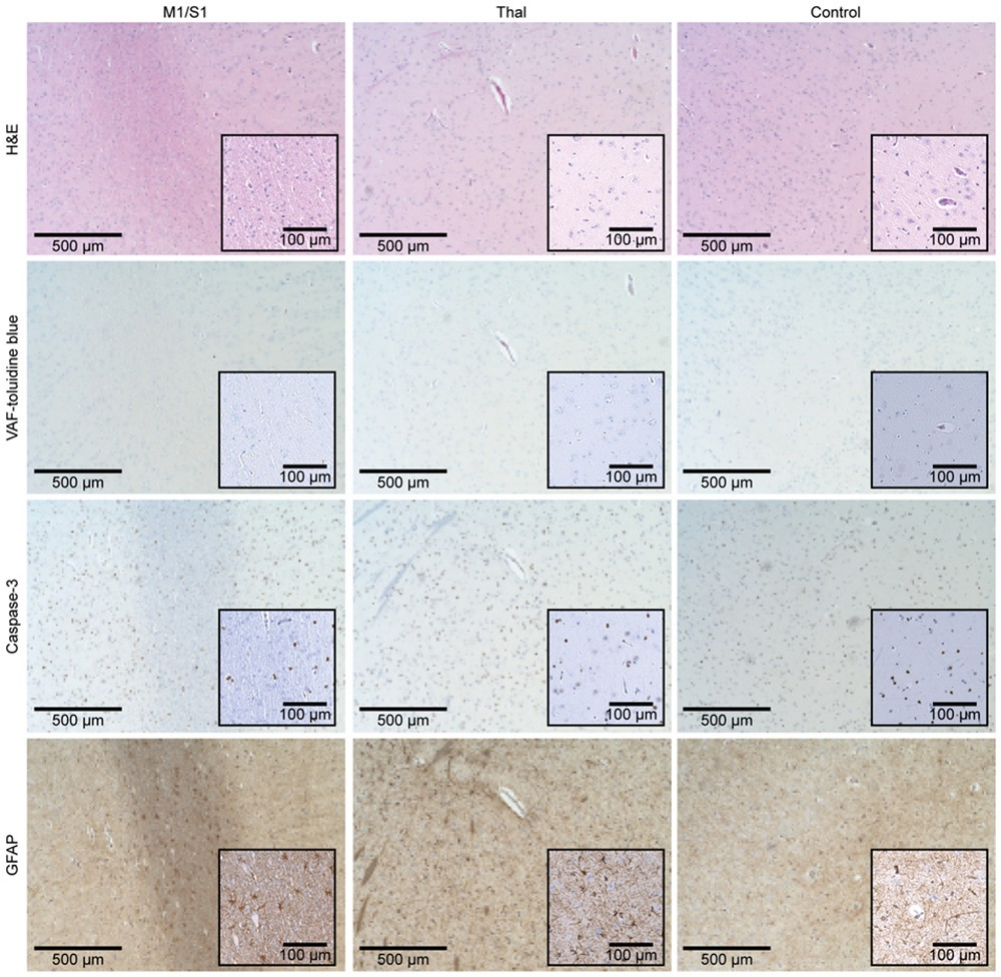
Exemplar histology results of sheep brain tissue (‘SH4’). The microscopic images (×40 magnification) of the respective tissue sampling location (M1/S1, Thal, and control site) are displayed according to the staining method (H&E, VAF-toluidine blue, caspase-3, and GFAP). Insets represent magnified images (×100 magnification).

We did not observe epileptographic EEG features (repetitive EEG spikes with voltage values that exceed 5 μV) during the acquisition of the resting-state EEGs before or after the FUS procedure (n = 4). Neither anatomical MRI nor contrast-enhanced MRI revealed presence of gross tissue damage or BBB disruption. During the post-sonication behavior monitoring period, all sheep demonstrated normal behavior without loss of appetite or weight loss. No signs of tissue damage (extravasation of erythrocytes, cell necrosis, inflammatory cells, ischemic neurons, apoptotic activity, glial infiltration, neurodegeneration) were detected with histological analysis of H&E, VAF-toluidine blue, caspase-3, and GFAP staining. Fig 9 shows exemplar microscopic images obtained from ‘SH4’.

## Discussion

Bimodal effects (i.e., excitation and suppression) of transcranial FUS in the modulation of the sensorimotor cortex and thalamus were investigated in a large animal model by evaluating the rate and magnitude of electrophysiological responses to a wide range of sonication parameters. To our knowledge, this is the first study to examine the effects of different pulsing schemes of FUS on the production of modulatory effects in a large animal model. The safety profile of repeated sonication sessions was also evaluated through behavioral monitoring and histological examination.

### Examination of excitatory effects

Response rates varied across the excitatory sonication parameters used in this study. The EMG response rates obtained from the hind leg contralateral to sonication (of both the M1 and thalamus) were higher compared to the responses from the hind leg ipsilateral to sonication. This finding suggests region-specific selective stimulation of the targeted brain areas. We also noted that responses from the hind leg ipsilateral to sonication were observed, although much less frequently. This finding shares similar features with those of previous TMS studies, in which stimulation of one hemisphere resulted in co-activation of the bilateral motor evoked potentials (MEPs) [77] or somatosensory responses [78]. We conjecture that existence of strong interhemispheric functional connections between the sensorimotor cortices [77, 78] and concurrent co-activation of bilateral efferent pathways, being extensively mediated by white matter connectivity between the motor areas [79, 80], may contribute to the presence of responses from both hind legs. Also, the presence of EMG responses elicited by FUS sonication to the thalamus suggests that thalamic stimulation ramifies activation of efferent motor units in the periphery. This finding agrees well with a previous study, in which motor thalamic electrical stimulation in non-human primates generated movements from various peripheral muscles, including the leg, as well as electrical responses from cortical motor areas [81].

The maximum response rate observed in the present study was on the order of 12.4%, measured from the contralateral hind leg when the parameter set EP17 (0.5 ms tone-burst duration, 70% DC, 200 ms sonication duration at I_a_ of 11.1 W/cm^2^ I_spta_; spatial-peak temporal-average intensity) was used to stimulate the M1. This response rate is much lower than those measured in our previous rodent studies on stimulating the M1 to elicit tail movement [30], but is comparable to (and even slightly higher than) observations by Mehić et al. [82], in which the motor response to acoustic stimulation was examined in mice. We speculate that differences in study design, including the type/depth/maintenance of anesthesia, choice of species, and stimulation parameters, might have contributed to this discrepancy. It is also possible that motion-related shifts in focal position (caused by breathing/ventilation) and skull-mediated misalignment of the acoustic focus may have led to inconsistencies in targeting of the selected brain area. Further study with use of a head fixating device [83] for sheep or conjunctional use of numerical simulation to predict the location of the acoustic focus [47] may reduce these experimental confounders. Interestingly, according to the Deblieck and colleagues [84], even well-established TMS protocols have significant variabilities in stimulatory efficiencies originating from many factors, such as cortical target, coil geometry/orientation and stimulation parameters.

When excitatory response rates were compared across varying sonication parameters (DC, tone-burst duration, and I_a_), the use of 70% DC yielded a significantly higher response rate than the use of 30 and 50% DCs, as well as continuous sonication (i.e., 100% DC) in stimulating the M1 (Fig 3A). The use of 100% DC showed a significantly reduced response rate, even when compared to those from 30 and 50% DCs. The same was true for thalamic stimulation, with 70% DC resulting in a higher response rate. In terms of tone-burst duration (Fig 3B), the use of 0.5 ms tone-burst duration showed a higher response rate compared to other tone-burst durations in both M1 and thalamic stimulation. These results are similar to our findings in previous investigations using rats, in which pulsed administration of FUS yielded a higher response rate compared to continuous sonication [30]; however, a discrepancy exists in that the use of 70% DC resulted in the highest EMG response rates whereas our rodent study showed the use of 50% DC elicited tail movement at the lowest FUS intensity. Direct comparisons between the two studies are difficult due to the differences in experimental designs and used animal species. Further studies identifying optimal sonication parameters for acoustic neuromodulation will benefit the ultimate translation of the technique to humans. Although the present study was not designed to examine the role of PRF on stimulation, the 70% DC condition, involving a higher PRF than other conditions, yielded the highest response rate across the tested range of DCs. This observation coincides well with a previous investigation by King et al. (2013) [28] showing that increasing PRF also increases the response rate.

We found that the use of 15.8 W/cm^2^ I_sppa_ generated a higher response rate than the use of a stronger I_a_ (18.2 W/cm^2^ I_sppa_) although this difference was not statistically significant in thalamic stimulation (Fig 3C). We originally anticipated that a higher I_a_ would increase the response rate, according to previous investigations by ourselves and others [28, 30, 82, 85] as well as numerical modeling [86]. However, our present findings suggest that, once stimulation exceeds a certain acoustic intensity, further increases in intensity may not necessarily benefit the stimulation efficiency. It is important to note that increasing the acoustic intensity without changing the focal geometry would also expose a greater volume of brain tissue to the intensity level for stimulation, which in turn may introduce confounders when determining the stimulation efficiency. The use of a wide range of acoustic intensities, in combination with exploration of a wider spectrum of sonication parameters and focal shapes, may provide more information on the relationship between acoustic intensity and stimulation efficiency.

In the examination of onset latencies of the EMG elicited by FUS (Fig 4), we identified a wide range of latencies that were mostly longer than ~25 ms, which was similar to the observations from previous small and large animal studies [28, 31, 72]. The presence of a broad onset latency distribution of EMG elicited by FUS, unlike the predictable and fixed latency of ~25 ms seen in TMS-elicited responses [87, 88], indicates that the mechanism of acoustic neural stimulation is likely different from that of TMS. Interestingly, a recent numerical model named ‘neuronal intramembrane cavitation excitation (NICE)’ suggests that FUS-mediated tissue-level brain stimulation is a ramification of a group of neuronal cells (e.g., excitatory regular spiking neurons, inhibitory fast spiking neurons, and inhibitory low-threshold spiking neurons) that respond to acoustic pressure waves with additional latencies for the accumulation of electrical charge across the neural membrane.

In the present data set, the onset latencies were detected most frequently at 50–75 ms after both M1 and thalamic stimulation, which was similar to our previous investigation in sheep (57.1 ± 48.8 ms [31]). The averaged time-locked contralateral EMGs elicited by FUS applied to either the M1 or the thalamus at 70% DC showed elevated magnitudes in the range of 100–190 ms when compared to the control EMG signals (Fig 5). Although we could not find any studies that measured the EMG latencies of brain stimulation in sheep (with the exception of our previous study [31]), we anticipated a latency of 35 ms or more from the sheep hind leg. This estimate considered additional limb distance (~50 cm) compared to human hands (EMG latency of ~25 ms [89]) and the use of anesthesia which is known to further increase the latency [90]. While the mechanisms underlying the latency distribution remain unknown, our observations suggest that the EMG responses stem from cortical excitation.

The incidence of EMG responses that occur shortly after acoustic stimulation (25 ms or shorter latency) has also been observed in previous rodent studies [28, 72], indicating that these short-latency responses may be associated with a reflex-type response (e.g., startle reflex seen in rodents [74]). The presence of short-latency responses (25 ms or shorter) suggests that, in spite of careful assessment of the anesthetic state of the animal, potential confounding effects from auditory/nociceptive or reflex-type startle associated with insufficient level of anesthesia may not be completely ruled out in the present study. While no sound was audible during suppressive sonication, we noticed audible sound (buzzing) during several excitatory sonication sessions depending on the placement of the coupling hydrogel between the FUS transducer and skin. This observation bears similarity to a previous human study [40]. We conjecture that the sound was created by the vibration of the coupling hydrogel on the skin due to the administration of FUS in an audible frequency range (i.e., PRF). However, the majority of EMG responses showed delayed latencies (>25 ms), which would not be explained by the startle reflex alone [75]. Investigations on measuring cell-level responses to the acoustic stimulation are urgently needed to elucidate the exact mechanism.

The motor response of the EMGs elicited by sonication of the M1 or the thalamus was not accompanied by overt limb movements, which were detected in previous small animal studies [4, 22, 28, 73, 82, 85]. The absence of overt movement is similar to previous M1 stimulation studies using FUS in large animal models (sheep) [31] and humans (increased fMRI-BOLD activation of the M1 area during thumb tapping task) [42]. We conjecture that the absence of elicited movements may be attributed to insufficient recruitment of motor units at the periphery due to the small size of the acoustic focus [31] and/or due to confounding effects from the type and depth of anesthesia [28, 73, 85].

Most of the previous studies conducted on anesthetized animals showed significant variability in the response to stimulation depending on the type and depth of anesthesia [73, 91]. To examine response to stimulation without the confounding effects from anesthesia, experiments in an awake setting are required, and several recent studies on non-human primates and human subjects have started to demonstrate the feasibility of FUS in brain stimulation without the use of anesthesia [33, 34, 37–43]. To enable studies in an unanesthetized, freely-moving large animal model (sheep), development of a wearable FUS setup [38, 43, 73] would be critical.

### Examination of suppressive effects

Across the different suppressive sonication parameters (SP1–SP8) and sonication targets (the S1 and thalamus), the group-averaged, normalized SEP peak magnitude acquired from the contralateral side from electrical stimulation of the hind leg (Fig 6) showed that a few sonication parameter sets (i.e., SP1 and SP3; tone-burst duration of 0.5 ms and I_sppa_ of 5.4 W/cm^2^; acquired at 3 and 5% DCs) suppressed the SEP magnitude (18–35% reduction). The reduced SEP magnitude persisted during the post-sonication period with the use of the SP1 parameter (‘P1’ segment, in sonication of both the S1 and thalamus) and the SP3 parameter (‘P1’ segment, in sonication of the S1). Our findings are comparable to the previous study, in which the reduction of visual evoked potential (VEP) magnitude (up to 13%) was observed after delivery of suppressive sonication to the visual cortex of rats during passive photic stimulation with use of 0.5 ms tone-burst duration and 5% DC [29]. The existence of a group of parameter sets for the suppression may be related to the differential responses to the temporal features and magnitude of acoustic pressure wave depending on the subtypes of neuronal cells, for example, as addressed by the NICE model [86]. Further investigation is needed, including measurement of electrophysiological responses of neurons cultured *in vitro*.

The suppressive effects were reversible, as the SEP magnitude induced by 2 min exposure to FUS was restored within 5 min after sonication (Fig 7). Despite differences in experimental design and choice of species, our findings bear similarity with work by Dallapiazza and colleagues [32], where low-intensity FUS applied to VPL thalamic nucleus suppressed SEP amplitude in a swine model. The retrospective temporal analysis of the SEPs acquired during the FUS sessions using the effective sonication parameters (i.e., SP1 and SP3) showed significant reduction of the SEP amplitudes within the temporal window of 40–60 ms (across the time segments of ‘F1–P5’) compared to the control condition. According to Nakamura et al. (2017) [92], the ovine SEP component at ~50 ms (e.g., P_50_) after stimulation could be mainly determined by sensory nerve conduction velocity and synaptic connectivity in subcortical and cortical pathways. Therefore, the reduced SEP amplitude within the 40–60 ms window may indicate the temporary disruption of synaptic connectivity by FUS-mediated suppression of either subcortical (thalamic) or cortical pathways. However, as studies measuring SEP in sheep are limited, further characterization of ovine SEP is warranted to understand the physiological representation of each SEP component in sheep.

For the assessment of suppressive effects, we applied pulsed sonication (at DCs of 3 or 5%) continuously for the duration of 2 min (i.e., F1 and F2) while delivering 50-μs electrical stimulations to the hind leg every 500 ms to elicit the SEP, with discrete EEG recording time windows. Therefore, it is conceivable that the portion of sonication given between the repeated electrical stimuli might have contributed to the observed suppression of SEP. FUS given at a much shorter time duration in a time-locked fashion, e.g., before, during, and after the electrical stimulation, will help to elucidate parameter-specific effects of FUS on neuronal tissue excitability.

### Estimation of potential thermal effects, post-sonication behavior monitoring, and histological assessment

From analysis of potential temperature increase due to sonication, both theoretical (Fig 8) and actual measurements using a tissue phantom revealed that temperature change at the focus was not detected at the highest energy deposition level. This supports that neuromodulatory effects are not related to sonication-mediated alterations in tissue temperature. No epileptographic features was observed during the resting-state EEGs, and behavior of all sheep was normal during the post-FUS survival period. No histological signs of tissue damage were detected, suggesting that repeated sonication sessions can be safely administered using the given experimental procedures.

We also note that continuous, short burst sonication (60, 100, and 140 ms durations used in excitatory sonication; Table 1), with up to 18.2 W/cm^2^ *in situ* I_spta_, was used safely in sheep. This intensity is much higher than the level used in our previous sheep studies [31], in which minor microhemorrhages were observed in the primary visual areas that were exposed to highly repetitive FUS sonication administered every second (ISI = 1 s) more than 500 times for EEG measurements at lower intensities (6.6–10.5 W/cm^2^ I_sppa_). Although the mechanism of tissue damage *via* highly repetitive excitatory FUS is difficult to ascertain, we previously hypothesized that longer time intervals between sonication trials (ISI) would lower the risk of tissue damage [31], and indeed, the time interval of 5 s introduced in the present study did not show any evidence of tissue damage.

### Potential mechanisms

Although there is a recent study suggesting that thermal effects from FUS may serve as a dominant biophysical mechanism behind neuro-inhibition [93], the mechanism underlying FUS-mediated neuromodulation remains unclear. We believe non-thermal, mechanical effects of the acoustic pressure waves play a major role in the observed phenomena, as demonstrated by the negligible temperature increase from sonication. Indeed, Plaksin and colleagues [94] proposed a numerical model in which the mechanical pressure wave of sonication can cause changes in neuronal membrane capacitance (*via* intramembrane cavitation) and subsequent transmembrane ion currents [94], which leads to excitation of the neural cells [95]. Further consideration of T-type (low-voltage activated) calcium channels and cell-type-selective mechanisms (such as interneurons or pyramidal neurons) was used to predict both excitation and suppression of neural circuits (referred to as the NICE model) [86].

The model’s prediction showed good agreement with our results, in which the uses of lower DCs (3 and 5%) yielded a suppressive effect and the uses of higher DCs (50 and 70%) elicited an excitatory effect. In particular, the NICE model predicted the existence of an optimum DC of 70%, which is well matched with our results. However, a discrepancy was observed in the use of 100% DC (i.e., continuous sonication). Unlike the prediction by the NICE model, our result showed that use of 100% DC decreased the response rate. Further investigation is needed to elucidate the cause of this discrepancy, including the potential involvement of diverse mechanisms in the observed neuromodulation. For example, another plausible mechanism is the hypothesis that the mechanical oscillation induced by FUS may generate a vibration motion of the bilayer membrane, which alters the state of mechanosensitive ion channels embedded within the cellular membrane and subsequently causes transmembrane current flow and cellular discharge [96, 97]. It is also conceivable that the mechanosensitive glial system [98] may be activated by FUS, thus contributing to neuromodulatory effects.

The exact neurobiological origin of FUS neuromodulation is still unknown, and considering the wide variation in neural cell types (excitatory/inhibitory neurons, local synaptic/corticospinal cells, or glia) with varying piezoelectric properties and spatial orientation in the sonicated brain tissue, further investigation is warranted in order to better understand the cellular and tissue-level neural responses to acoustic pressure waves. Adoption of experimental procedures that are analogous to the paired-pulse TMS (ppTMS) protocol, in which administration of paired TMS pulses are used to examine the presence of intracortical inhibition and facilitation [99, 100], may also be conducive to elucidating the tissue-level mechanism behind the modulatory effect. Recently, Legon and colleagues have shown that concurrent application of TMS and FUS to the motor cortical area in humans inhibited the amplitude of single-pulse MEPs and attenuated intracortical facilitation, while FUS did not affect intracortical inhibition [44].

### Technical limitations of the study

Although we have demonstrated region-specific modulation of brain activity using FUS, we also note that there are several technical limitations of this study. First, the image-guidance and sonication used in the study did not have sufficient spatial accuracy to discern the VPL and VL in an ovine model, given the FRE of 5.8 ± 1.5 mm and the FUS focal dimensions of 3 mm in diameter and 13 mm in length, creating a significant margin of error in sonication. The main source of FRE was ambiguity in anatomical features (eye, nose, and ear canal). In addition, the wool/lanolin prevented stable placement and attachment of the fiducial markers on the skin. The use of rigid markers implanted to the skull could improve the registration accuracy. Further aberration of the acoustic focus, due to presence of the skull and head motion from ventilation of the animal, inevitably contributed to additional error in sonication. We acknowledge the challenge of overcoming these confounders, and we believe the use of an arrayed FUS transducer with phase correction schemes [101, 102], operating at a higher frequency (for a smaller focal size), with elaborate numerical acoustic simulation will help to increase the spatial fidelity of sonication.

### Therapeutic potential

We have demonstrated that neuroimage-guided FUS delivered to specific cortical and thalamic regions of the brain is capable of selectively activating or suppressing corresponding neural circuits in a large animal model. Non-invasive and non-pharmacological modulation of the excitability of regional neuronal structures offers great potential for neurotherapeutics. Excitatory effects of sonication may be utilized for enhancing regional excitability for various therapeutic applications such as neurorehabilitation [103]. The transient neuromodulatory effects of FUS shown in this study, especially in terms of suppressive effects, could be used to study functional connectivity through non-invasive and safe functional brain mapping of both cortical and deep brain neuroanatomies and could be used before irreversible functional neurosurgical procedures, including tissue ablation using high intensity ultrasound [16–18]. Suppressive FUS sonication would also be useful in stabilizing states of abnormal hyper-excitability of the brain, such as in seizure disorders, and we previously reported that suppressive FUS to the thalamic area of an acute epilepsy rat model reduces seizure activity [65]. Moreover, we and others demonstrated the durable change of cortical activity beyond the duration of sonication in animal models [35, 36, 104]. Further investigation regarding this long-term modulation of brain activity, with potential for inducing neuroplasticity, is crucial for determining the therapeutic effects of FUS-mediated brain stimulation [105].

## Supporting information

S1 Fig Example of the recorded EMG and EEG profiles without FUS sonication. (A) EMG signal (low-pass filtered using threshold of 200 Hz) obtained from the gastrocnemius of the right hind limb showed three signal bursts (marked by red arrows; indicating the first negative peak) elicited by superficial mechanical stimulation of the corresponding leg nerve. The blue dashed line indicates the timing of stimulation onset. (B) EMG signal (low-pass filter of 30 Hz high cut-off) from the time segment marked by the bracket in (A). (C) EEG SEP signal (bandpass filtered at 0.5–200 Hz) induced by electrical stimulation of the contralateral hind leg. A negative peak (N_40_) and positive peak (P_50_) were detected at ~40 ms and ~50 ms, respectively.
(PDF)

S2 Fig Experimental timeline from arrival to sacrifice across ten animals (‘SH1’–‘SH10’). (PDF)

S3 Fig Time-locked contralateral EMG from M1/thalamic stimulation. The averaged EMG signals were obtained from the use of 70% DC (i.e., EP17–EP24). The data from stimulation of the M1 and thalamus is displayed in the blue and green lines, respectively. The data obtained in the absence of sonication is plotted in the black line (labeled as ‘No FUS’). The baseline signal drift/offset was removed from all individual EMG data with respect to FUS onset. The colored bars indicate regions of significant differences (p < 0.01, one-tailed *t*-test) in the amplitude obtained from M1 (in blue) and thalamic (in green) stimulation compared to the amplitude obtained when FUS was not given (i.e., ‘No FUS’). Dashed lines indicate the onset timing of FUS sonication, and thick solid black bars represent the duration of sonication.
(PDF)

S1 Table Excitation sonication results obtained from contralateral EMG in the M1 stimulations. T: total number of sonications, P: number of elicited responses, R: response rate (%).
(DOCX)

S2 Table Excitation sonication results obtained from ipsilateral EMG in the M1 stimulations. T: total number of sonications, P: number of elicited responses, R: response rate (%).
(DOCX)

S3 Table Excitation sonication results obtained from contralateral EMG in the thalamic stimulations. T: total number of sonications, P: number of elicited responses, R: response rate (%).
(DOCX)

S4 Table Excitation sonication results obtained from ipsilateral EMG in the thalamic stimulations. T: total number of sonications, P: number of elicited responses, R: response rate (%).
(DOCX)
