# Supplementary material for: Effects of sonication parameters on transcranial focused ultrasound brain stimulation in an ovine model
Source: PLoS One. 2019 Oct 24;14(10):e0224311. doi: 10.1371/journal.pone.0224311 (PMC6812789; doi:10.1371/journal.pone.0224311)
Supplement: S1 Fig — (A) EMG signal (low-pass filtered using threshold of 200 Hz) obtained from the gastrocnemius of the right hind limb showed three signal bursts (marked by red arrows; indicating the first negative peak) elicited by superficial mechanical stimulation of the corresponding leg nerve. The blue dashed line indicates the timing of stimulation onset. (B) EMG signal (low-pass filter of 30 Hz high cut-off) from the time segment marked by the bracket in (A). (C) EEG SEP signal (bandpass filtered at 0.5–200 Hz) induced by electrical stimulation of the contralateral hind leg. A negative peak (N40) and positive peak (P50) were detected at ~40 ms and ~50 ms, respectively. (PDF) [file pone.0224311.s001.pdf]

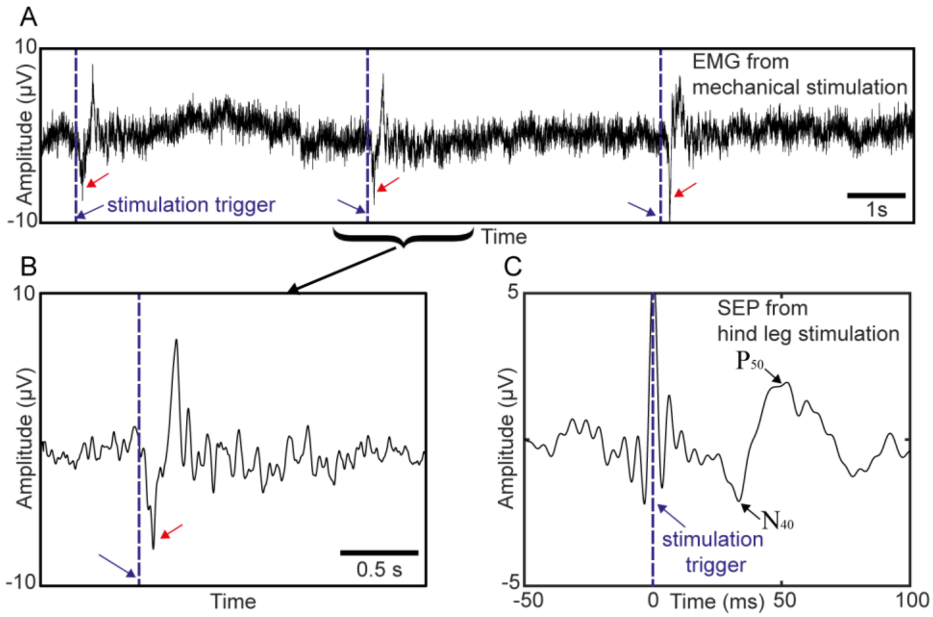

S1 Fig. Example of the recorded EMG and EEG profiles without FUS sonication. (A) EMG signal (low-pass filtered using threshold of 200 Hz) obtained from the gastrocnemius of the right hind limb showed three signal bursts (marked by red arrows; indicating the first negative peak) elicited by superficial mechanical stimulation of the corresponding leg nerve. The blue dashed line indicates the timing of stimulation onset. (B) EMG signal (low-pass filter of 30 Hz high cut-off) from the time segment marked by the bracket in (A). (C) EEG SEP signal (bandpass filtered at 0.5–200 Hz) induced by electrical stimulation of the contralateral hind leg. A negative peak (N_{40}) and positive peak (P_{50}) were detected at ~40 ms and ~50 ms, respectively.
