# Supplementary figures and images for: Effects of sonication parameters on transcranial focused ultrasound brain stimulation in an ovine model
Source: PLoS One. 2019 Oct 24;14(10):e0224311. doi: 10.1371/journal.pone.0224311 (PMC6812789; doi:10.1371/journal.pone.0224311)

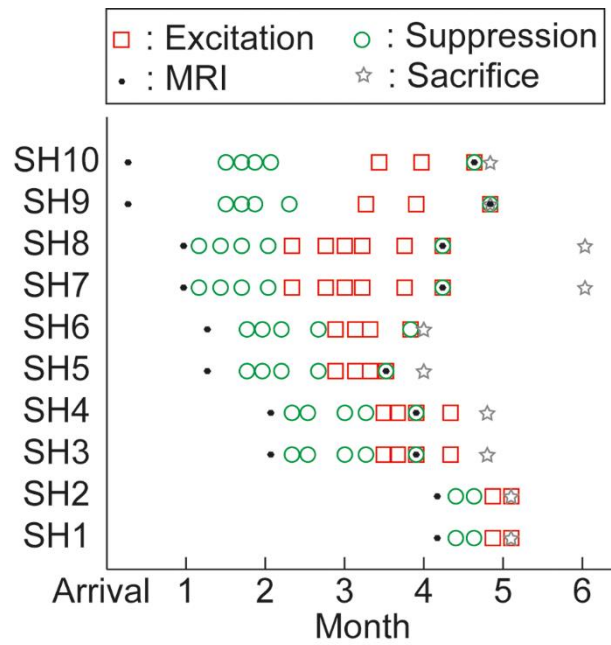

**S2 Fig. Experimental timeline from arrival to sacrifice across ten animals (‘SH1’–‘SH10’).**

Supplement: S2 Fig — (PDF) [file pone.0224311.s002.pdf]
