# Supplementary material for: Effects of sonication parameters on transcranial focused ultrasound brain stimulation in an ovine model
Source: PLoS One. 2019 Oct 24;14(10):e0224311. doi: 10.1371/journal.pone.0224311 (PMC6812789; doi:10.1371/journal.pone.0224311)
Supplement: S3 Fig — The averaged EMG signals were obtained from the use of 70% DC (i.e., EP17–EP24). The data from stimulation of the M1 and thalamus is displayed in the blue and green lines, respectively. The data obtained in the absence of sonication is plotted in the black line (labeled as ‘No FUS’). The baseline signal drift/offset was removed from all individual EMG data with respect to FUS onset. The colored bars indicate regions of significant differences (p < 0.01, one-tailed t-test) in the amplitude obtained from M1 (in blue) and thalamic (in green) stimulation compared to the amplitude obtained when FUS was not given (i.e., ‘No FUS’). Dashed lines indicate the onset timing of FUS sonication, and thick solid black bars represent the duration of sonication. (PDF) [file pone.0224311.s003.pdf]

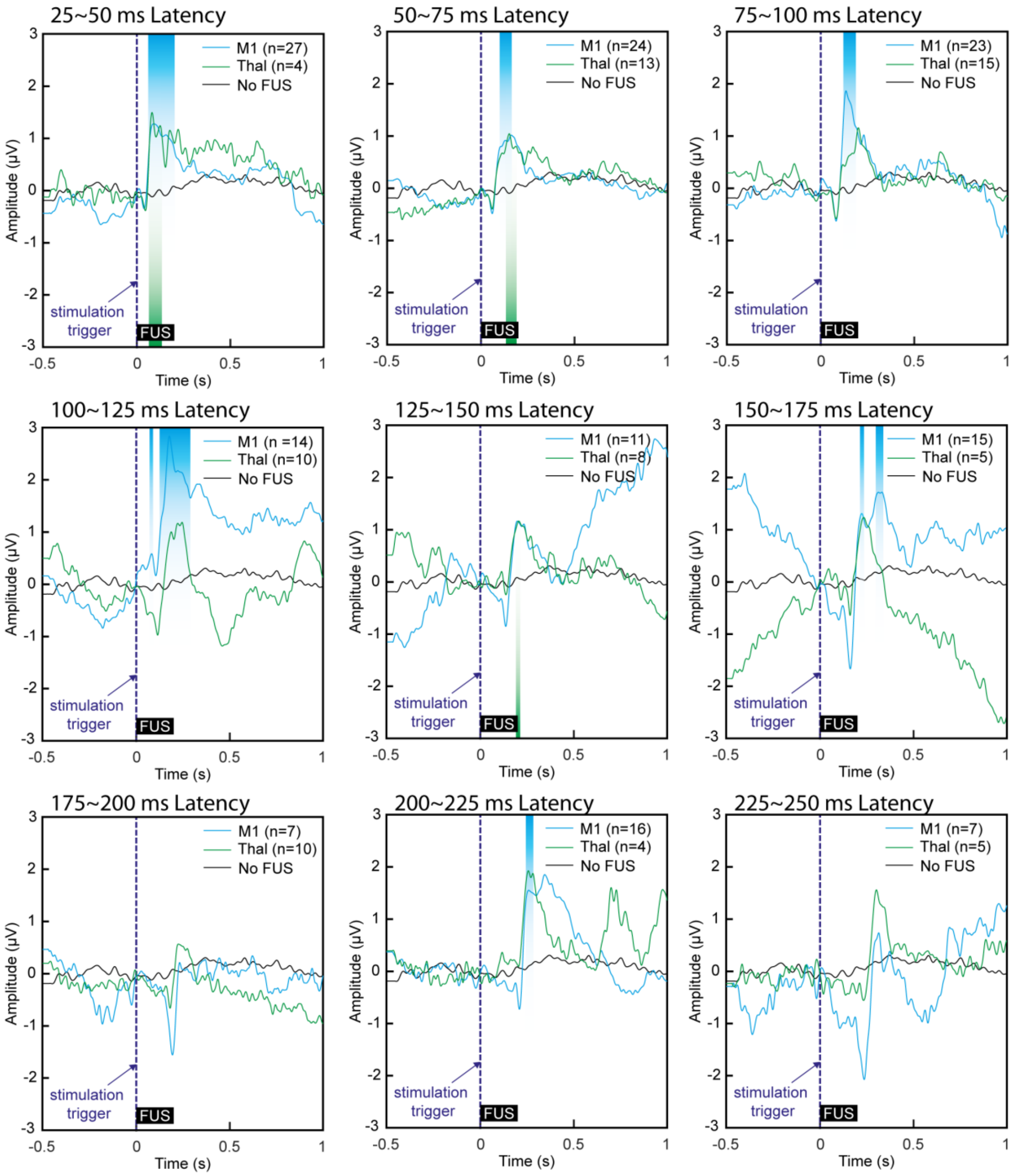

S3 Fig. Time-locked contralateral EMG from M1/thalamic stimulation. The averaged EMG signals were obtained from the use of 70% DC (i.e., EP17–EP24). The data from stimulation of the M1 and thalamus is displayed in the blue and green lines, respectively. The data obtained in the absence of sonication is plotted in the black line (labeled as 'No FUS'). The baseline signal drift/offset was removed from all individual EMG data with respect to FUS onset. The colored bars indicate regions of significant differences ($p < 0.01$, one-tailed *t*-test) in the amplitude obtained from M1 (in blue) and thalamic (in green) stimulation compared to the amplitude obtained when FUS was not given (i.e., 'No FUS'). Dashed lines indicate the onset timing of FUS sonication, and thick solid black bars represent the duration of sonication.
