# Supplementary material for: Effects of sonication parameters on transcranial focused ultrasound brain stimulation in an ovine model
Source: PLoS One. 2019 Oct 24;14(10):e0224311. doi: 10.1371/journal.pone.0224311 (PMC6812789; doi:10.1371/journal.pone.0224311)
Supplement: S2 Table — T: total number of sonications, P: number of elicited responses, R: response rate (%). (DOCX) [file pone.0224311.s005.docx]

**S2 Table. Excitation sonication results obtained from ipsilateral EMG in the M1 stimulations.** T: total number of sonications, P: number of elicited responses, R: response rate (%).

| Para | SH1 | | | SH2 | | | SH3 | | | SH4 | | | SH5 | | | SH6 | | | SH7 | | | SH8 | | | SH9 | | | SH10 | | |
| --- | --- | --- | --- | --- | --- | --- | --- | --- | --- | --- | --- | --- | --- | --- | --- | --- | --- | --- | --- | --- | --- | --- | --- | --- | --- | --- | --- | --- | --- | --- |
| set ID | T | P | R | T | P | R | T | P | R | T | P | R | T | P | R | T | P | R | T | P | R | T | P | R | T | P | R | T | P | R |
| EP1 | - | - | - | - | - | - | 60 | 1 | 1.7 | 80 | 2 | 2.5 | 40 | 0 | 0.0 | 40 | 0 | 0.0 | 20 | 2 | 10.0 | 20 | 0 | 0.0 | 20 | 1 | 5.0 | 20 | 0 | 0.0 |
| EP2 | - | - | - | - | - | - | 60 | 1 | 1.7 | 80 | 1 | 1.3 | 40 | 0 | 0.0 | 40 | 1 | 2.5 | 20 | 0 | 0.0 | 20 | 0 | 0.0 | 20 | 0 | 0.0 | 20 | 0 | 0.0 |
| EP3 | - | - | - | - | - | - | 60 | 0 | 0.0 | 80 | 0 | 0.0 | 40 | 0 | 0.0 | 40 | 0 | 0.0 | 20 | 0 | 0.0 | 20 | 0 | 0.0 | 20 | 0 | 0.0 | 20 | 0 | 0.0 |
| EP4 | - | - | - | - | - | - | 60 | 0 | 0.0 | 80 | 0 | 0.0 | 40 | 1 | 2.5 | 40 | 3 | 7.5 | 20 | 1 | 5.0 | 20 | 0 | 0.0 | 20 | 0 | 0.0 | 20 | 0 | 0.0 |
| EP5 | - | - | - | - | - | - | 60 | 3 | 5.0 | 80 | 0 | 0.0 | 40 | 0 | 0.0 | 40 | 1 | 2.5 | 20 | 1 | 5.0 | 20 | 0 | 0.0 | 20 | 0 | 0.0 | 20 | 1 | 5.0 |
| EP6 | - | - | - | - | - | - | 60 | 0 | 0.0 | 80 | 2 | 2.5 | 40 | 0 | 0.0 | 40 | 0 | 0.0 | 20 | 0 | 0.0 | 20 | 0 | 0.0 | 20 | 0 | 0.0 | 20 | 0 | 0.0 |
| EP7 | - | - | - | - | - | - | 60 | 2 | 3.3 | 80 | 0 | 0.0 | 40 | 0 | 0.0 | 40 | 0 | 0.0 | 20 | 0 | 0.0 | 20 | 0 | 0.0 | 20 | 0 | 0.0 | 20 | 0 | 0.0 |
| EP8 | - | - | - | - | - | - | 60 | 1 | 1.7 | 80 | 2 | 2.5 | 40 | 0 | 0.0 | 40 | 1 | 2.5 | 20 | 0 | 0.0 | 20 | 0 | 0.0 | 20 | 0 | 0.0 | 20 | 0 | 0.0 |
| EP9 | - | - | - | - | - | - | 60 | 0 | 0.0 | 80 | 3 | 3.8 | 40 | 0 | 0.0 | 40 | 0 | 0.0 | 20 | 0 | 0.0 | 20 | 0 | 0.0 | 20 | 1 | 5.0 | 20 | 0 | 0.0 |
| EP10 | - | - | - | - | - | - | 60 | 1 | 1.7 | 80 | 3 | 3.8 | 40 | 0 | 0.0 | 40 | 1 | 2.5 | 20 | 0 | 0.0 | 20 | 0 | 0.0 | 20 | 1 | 5.0 | 20 | 1 | 5.0 |
| EP11 | 20 | 1 | 5.0 | - | - | - | 60 | 0 | 0.0 | 80 | 2 | 2.5 | 40 | 0 | 0.0 | 40 | 1 | 2.5 | 20 | 2 | 10.0 | 20 | 1 | 5.0 | 20 | 0 | 0.0 | 20 | 0 | 0.0 |
| EP12 | - | - | - | 20 | 2 | 10.0 | 60 | 1 | 1.7 | 80 | 3 | 3.8 | 40 | 1 | 2.5 | 40 | 0 | 0.0 | 20 | 1 | 5.0 | 20 | 0 | 0.0 | 20 | 0 | 0.0 | 20 | 0 | 0.0 |
| EP13 | 20 | 0 | 0.0 | - | - | - | 60 | 2 | 3.3 | 80 | 3 | 3.8 | 40 | 0 | 0.0 | 40 | 1 | 2.5 | 20 | 0 | 0.0 | 20 | 0 | 0.0 | 20 | 0 | 0.0 | 20 | 0 | 0.0 |
| EP14 | 60 | 1 | 1.7 | - | - | - | 60 | 0 | 0.0 | 80 | 1 | 1.3 | 40 | 1 | 2.5 | 40 | 2 | 5.0 | 20 | 0 | 0.0 | 20 | 0 | 0.0 | 20 | 0 | 0.0 | 20 | 0 | 0.0 |
| EP15 | - | - | - | - | - | - | 60 | 4 | 6.7 | 80 | 1 | 1.3 | 40 | 0 | 0.0 | 40 | 0 | 0.0 | 20 | 0 | 0.0 | 20 | 0 | 0.0 | 20 | 0 | 0.0 | 20 | 0 | 0.0 |
| EP16 | - | - | - | - | - | - | 60 | 0 | 0.0 | 80 | 2 | 2.5 | 40 | 2 | 5.0 | 40 | 2 | 5.0 | 20 | 2 | 10.0 | 20 | 0 | 0.0 | 20 | 0 | 0.0 | 20 | 0 | 0.0 |
| EP17 | - | - | - | - | - | - | 60 | 1 | 1.7 | 80 | 2 | 2.5 | 40 | 2 | 5.0 | 40 | 0 | 0.0 | 20 | 2 | 10.0 | 20 | 0 | 0.0 | 20 | 1 | 5.0 | 20 | 0 | 0.0 |
| EP18 | - | - | - | - | - | - | 60 | 2 | 3.3 | 80 | 2 | 2.5 | 40 | 4 | 10.0 | 40 | 0 | 0.0 | 20 | 1 | 5.0 | 20 | 1 | 5.0 | 20 | 0 | 0.0 | 20 | 0 | 0.0 |
| EP19 | - | - | - | - | - | - | 60 | 1 | 1.7 | 80 | 4 | 5.0 | 40 | 1 | 2.5 | 40 | 2 | 5.0 | 20 | 2 | 10.0 | 20 | 3 | 15.0 | 20 | 0 | 0.0 | 20 | 0 | 0.0 |
| EP20 | - | - | - | - | - | - | 60 | 3 | 5.0 | 80 | 1 | 1.3 | 40 | 1 | 2.5 | 40 | 1 | 2.5 | 20 | 3 | 15.0 | 20 | 1 | 5.0 | 20 | 1 | 5.0 | 20 | 0 | 0.0 |
| EP21 | - | - | - | - | - | - | 60 | 2 | 3.3 | 80 | 3 | 3.8 | 40 | 1 | 2.5 | 40 | 0 | 0.0 | 20 | 0 | 0.0 | 20 | 0 | 0.0 | 20 | 0 | 0.0 | 20 | 1 | 5.0 |
| EP22 | - | - | - | 20 | 0 | 0.0 | 60 | 2 | 3.3 | 80 | 3 | 3.8 | 40 | 1 | 2.5 | 40 | 0 | 0.0 | 20 | 0 | 0.0 | 20 | 1 | 5.0 | 20 | 0 | 0.0 | 20 | 0 | 0.0 |
| EP23 | - | - | - | - | - | - | 60 | 0 | 0.0 | 80 | 1 | 1.3 | 40 | 2 | 5.0 | 40 | 1 | 2.5 | 20 | 0 | 0.0 | 20 | 0 | 0.0 | 20 | 0 | 0.0 | 20 | 1 | 5.0 |
| EP24 | - | - | - | - | - | - | 60 | 2 | 3.3 | 80 | 1 | 1.3 | 40 | 0 | 0.0 | 40 | 2 | 5.0 | 20 | 0 | 0.0 | 20 | 0 | 0.0 | 20 | 0 | 0.0 | 20 | 0 | 0.0 |
| EP25 | - | - | - | - | - | - | 40 | 1 | 2.5 | 40 | 0 | 0.0 | 40 | 0 | 0.0 | 40 | 0 | 0.0 | 20 | 0 | 0.0 | 20 | 1 | 5.0 | 20 | 0 | 0.0 | 20 | 0 | 0.0 |
| EP26 | - | - | - | - | - | - | 40 | 0 | 0.0 | 60 | 1 | 1.7 | 40 | 0 | 0.0 | 40 | 3 | 7.5 | 20 | 0 | 0.0 | 20 | 0 | 0.0 | 20 | 0 | 0.0 | 20 | 0 | 0.0 |
| EP27 | - | - | - | - | - | - | 40 | 0 | 0.0 | 40 | 0 | 0.0 | 40 | 0 | 0.0 | 40 | 2 | 5.0 | 20 | 0 | 0.0 | 20 | 0 | 0.0 | 20 | 0 | 0.0 | 20 | 0 | 0.0 |
| EP28 | - | - | - | - | - | - | 40 | 0 | 0.0 | 60 | 0 | 0.0 | 40 | 0 | 0.0 | 40 | 2 | 5.0 | 20 | 1 | 5.0 | 20 | 0 | 0.0 | 20 | 1 | 5.0 | 20 | 0 | 0.0 |
| EP29 | - | - | - | - | - | - | 40 | 0 | 0.0 | 20 | 0 | 0.0 | 40 | 1 | 2.5 | 40 | 1 | 2.5 | 20 | 0 | 0.0 | 20 | 0 | 0.0 | 20 | 0 | 0.0 | 20 | 1 | 5.0 |
| EP30 | - | - | - | - | - | - | 40 | 0 | 0.0 | 40 | 0 | 0.0 | 40 | 0 | 0.0 | 40 | 1 | 2.5 | 20 | 1 | 5.0 | 20 | 0 | 0.0 | 20 | 0 | 0.0 | 20 | 1 | 5.0 |
